# Semantic segmentation and quantitative analysis of tunnel cracks and water leakage using a TransUNet framework

**DOI:** 10.1371/journal.pone.0349175

**Published:** 2026-08-11

**Authors:** Xinjian Li, Qiaofeng Liu, Gang Yan, Qifeng Yu

**Affiliations:** 1 Business School, Guilin University of Electronic Technology, Guilin, China; 2 Guangxi Institute of Industrial Technology for Space-Time Information Co., Ltd, Nanning, China; 3 Guangxi Transport Vocational and Technical College, Nanning, China; 4 College of Transport and Communications, Shanghai Maritime University, Shanghai, China; Henan Polytechnic University, CHINA

## Abstract

As a vital component of structural health monitoring, the detection of cracks and water leakage in tunnel linings is essential for ensuring structural durability and operational safety. However, due to complex site conditions, such as non-uniform illumination, surface texture interference, and the slender, blurred nature of defects, traditional manual inspections and threshold-based algorithms often fail to provide reliable damage identification. To address these challenges, this study proposes an end-to-end semantic segmentation framework based on TransUNet. By integrating the local feature extraction of convolutional neural networks (CNNs) with the global dependency modeling of Transformers, the framework significantly enhances the characterization of multi-scale defects and boundary features. A comprehensive dataset comprising public benchmarks and real-world engineering images was developed using a standardized preprocessing and validation pipeline. The proposed method was systematically evaluated against state-of-the-art models like U-Net and DeepLabv3 + . Experimental results demonstrate that the TransUNet framework achieves an IoU of 71.57% for crack segmentation and a Precision of 91.51% for water leakage. Crucially for engineering applications, the geometric error for length and area measurements is maintained within 5%, while the inference latency remains under 200 ms. In terms of precision, boundary preservation, and geometric consistency, the proposed method shows clear advantages over the comparison models, while U‑Net exhibits stronger region overlap for water leakage detection. Overall, the method meets the requirements of offline inspection and near-real-time applications. This data-driven approach provides a robust technical foundation for tunnel defect detection and subsequent maintenance decision-making.

## 1. Introduction

With the continuous expansion of urban transportation networks and the sustained growth in infrastructure construction demands, tunnel engineering has become an important component of modern urban transportation systems and regional connectivity [1]. Tunnel structures, as vital infrastructure for underground space development and lifelines for transport, have seen rapid advancement in recent years [2]. However, owing to variations in construction era, surrounding rock conditions, construction techniques, and environmental loads, tunnel linings frequently exhibit multiple types of defects during long-term operation, including cracking, leakage, excessive deformation, and delamination [3,4]. These defects not only reduce the durability of the lining structure but may also lead to progressive deterioration of the lining, intensified seepage effects, and even local instability, thereby posing a substantial threat to tunnel operational safety [5,6]. Therefore, efficient identification, precise quantification, and intelligent diagnosis of tunnel defects are of great engineering significance for achieving scientific maintenance and life-extension management of tunnel facilities. Within the framework of sustainable transportation management, the structural integrity of tunnels is not merely a safety concern but a prerequisite for maintaining system resilience and minimizing the environmental and economic impacts caused by emergency repairs and traffic disruptions.

The development of tunnel defect detection technology has broadly progressed from manual inspections and traditional image processing methods to machine learning and deep learning techniques. Early detection primarily relied on experienced engineers identifying defects such as cracks and water leakage through on-site inspections or manual image analysis [7]. While intuitive, this approach suffered from limitations including low efficiency, high costs, and significant subjectivity. With the proliferation of image acquisition equipment, researchers began exploring semi-automated defect identification using conventional image processing techniques, such as threshold segmentation [8], edge detection [9,10], and region growing algorithms [11]. However, these methods are highly dependent on uniform illumination and grey scale differences and tend to fail when confronted with noise interference or indistinct defect characteristics. Addressing uneven illumination and noise interference, Liu et al. [12] noted that stains and shadows can disrupt crack connectivity, leading to the proposal of DeepCrack to enhance the integrity and continuity of slender cracks. Wu [13] combined an improved Retinex approach with deep networks, significantly improving crack separability and segmentation accuracy in low-illumination environments. Although traditional image processing methods have facilitated the transition from purely manual detection to automation, their reliance on manually set thresholds and rules limits their robustness in complex scenarios.

To overcome the limitations of manual inspection, such as low efficiency and high subjectivity, researchers have progressively introduced machine learning methods to conduct automated identification of tunnel defects. Medina and Llamas [14] proposed a crack detection algorithm based on Gabor filters, enabling direction-insensitive identification of crack edges and enhancing stability under complex lighting conditions. In real tunnel environments, research has also integrated feature pyramids with edge branches within the Mask R-CNN framework to enhance defect feature representation capabilities. Compared to traditional manual features, this approach demonstrates greater robustness under complex operational conditions [15]. Overall, while traditional machine learning models have achieved some success in crack detection tasks, they remain reliant on manually designed features and extensive model parameter tuning. This results in limited generalizability and often necessitates substantial training and validation samples across different scenarios. Consequently, model transferability and development costs are constrained [16,17].

With the rapid advancement of deep learning technology, tunnel defect detection has progressively shifted towards a data-driven, end-to-end recognition paradigm. Convolutional neural networks (CNNs) can automatically learn hierarchical features within images, thereby enabling high-precision detection and segmentation of defects such as cracks and water leakage [18]. To balance global semantic understanding with local detail representation, Tang et al. proposed TransFuse. By concurrently integrating CNN and Transformer branches, the model captures long-range dependencies and low-level spatial details within shallow structures [19]. In the domain of semantic segmentation, U-Net leverages its classic encoder-decoder architecture to deliver outstanding performance in medical image analysis and has been extensively adapted for structural crack detection [20]. DeepLabv3 + further integrates dilated convolutions with the Atrous Spatial Pyramid Pooling (ASPP) module, demonstrating significant advantages in multi-scale feature extraction and boundary identification [21]. Moreover, CrackFormer, proposed by Liu et al. [22], enhances the representation of thin, low-contrast cracks through a self-attention mechanism, effectively improving segmentation accuracy for fine-grained fcrissures. Although CNN-based segmentation models (such as U-Net, DeepLabv3+, etc.) have achieved certain results in identifying tunnel cracks and water leakage, their limited local receptive fields make it difficult to fully capture long-range dependencies. Particularly in scenarios with complex backgrounds, low-contrast defects, and blurred structural edges, these models are prone to incomplete segmentation or loss of boundary details. Furthermore, traditional CNN models exhibit shortcomings in modeling multi-scale feature consistency, struggling to simultaneously account for both global semantic information and fine-grained structural characteristics.

To overcome the limitations of CNNs, the introduction of Transformer architecture has provided a new breakthrough for image segmentation tasks. Non-local network established by Wang et al. [23] explicitly models long-range dependencies through non-local operators, effectively overcoming the local constraints of convolutions. Meanwhile, Visual Transformer (ViT) proposed by Dosovitskiy et al. [24] further demonstrates that pure Transformers, leveraging global self-attention, can compete effectively on visual tasks. The TransUNet model combines the local feature extraction capabilities of CNNs with the global modeling capabilities of Transformers. By embedding a ViT into the encoder of a U-Net, it achieves efficient fusion of object edges and texture information in complex scenes [25]. Recent studies have further advanced Transformer-based crack segmentation. For instance, CrackFormer embeds novel Transformer encoders into a SegNet-like architecture, achieving higher accuracy with substantially fewer computational resources [26]. LiteCrackSeg, a lightweight hybrid CNN–Transformer model, requires only 2.72 million parameters and achieves an inference speed of 56 frames per second, making it suitable for edge deployment [27]. Along this line of research, TransUNet has shown advantages over traditional CNN models in multi-scale feature representation, structural integrity restoration, and small-object detection, thereby offering improved accuracy and robustness for crack segmentation tasks. Existing research has demonstrated that Transformer-based networks exhibit significant performance advantages in applications such as crack detection, leakage identification, and concrete surface defect segmentation [28,29]. As Intelligent Transportation Systems (ITS) increasingly rely on multi-source data and AI-driven insights, the high-precision segmentation provided by TransUNet serves as a critical component for real-time traffic monitoring and long-term infrastructure health assessment, aligning with the shift towards smarter and more sustainable mobility systems.

Building upon the aforementioned research advances, this study proposed an automated detection method for tunnel cracks and water leakage based on TransUNet. By incorporating the Transformer self-attention mechanism into image segmentation networks, high-precision segmentation of tunnel defect areas is achieved. The experimental section selected U-Net and DeepLabv3+ as comparison models, comprehensively evaluating model performance through metrics including accuracy, recall, F1 score, and intersection-over-union (IoU). Ultimately, this study contributes to the advancement of structural health monitoring by providing a robust intelligent diagnosis tool that enhances structural safety assessment and infrastructure longevity. By facilitating predictive maintenance, this data-driven approach reduces the operational risks of tunnel facilities and strengthens the overall reliability of critical transportation infrastructure.

## 2. Methodology

### 2.1. Data collection and augmentation

This study uses a consolidated image dataset consisting of publicly available tunnel crack and water leakage datasets [30,31] and additional field-collected images from an in-service tunnel. The publicly available images were derived from open-source platforms and research datasets, while the field-collected images were used to further enrich the diversity of tunnel defect scenarios. The dataset contains images captured under different lighting conditions, shooting angles, and surface texture backgrounds, including various interference factors such as shadows, stains, reflections, and complex lining textures. These characteristics help reflect the diversity and complexity of tunnel cracks and water leakage defects in practical engineering environments.

**Fig 1:**
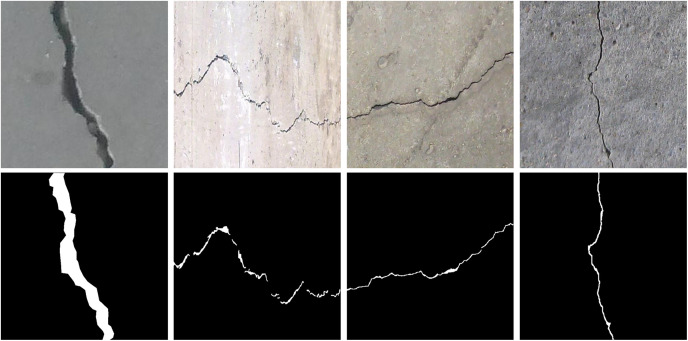
Examples of tunnel crack dataset images and corresponding masks (top: original images; bottom: ground‑truth images). The composite figure was prepared by the authors using images from the publicly available dataset reported in Ref. [30] and selected author-provided raw images available in S1 Data. The publicly available dataset images are distributed under a license compatible with publication under CC BY 4.0.

**Fig 2:**
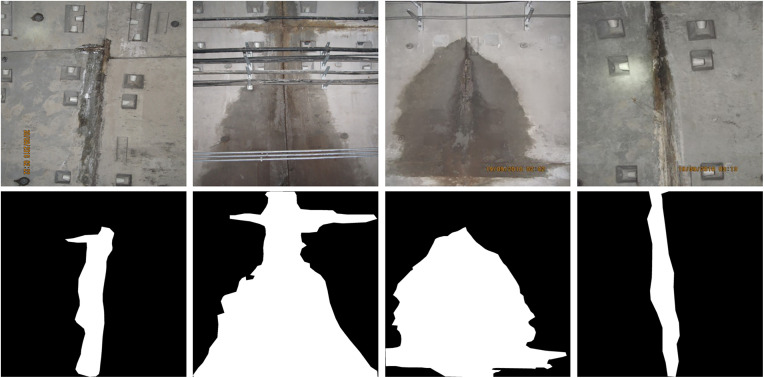
Examples of tunnel water leakage dataset images and corresponding masks (top: original images; bottom: ground‑truth images). The composite figure was prepared by the authors using images from the publicly available dataset reported in Ref. [31] and selected author-provided raw images available in S1 Data. The publicly available dataset images are distributed under a license compatible with publication under CC BY 4.0.

The consolidated dataset comprises approximately 200 crack images and 300 water leakage images. Crack samples predominantly exhibit elongated linear or branched structures, with some containing multiple parallel or intersecting fissures. Water leakage samples encompass diverse morphologies including punctate, linear, and patchy patterns, alongside areas displaying reflections, shadows, and water stain diffusion. Among these images, approximately 70% were obtained from publicly available datasets [30,31], while the remaining 30% were collected from an in-service tunnel. Pixel-wise annotations were generated using LabelMe and Roboflow Annotate following a standardized annotation workflow. Specifically, trained annotators first identified the visible defect regions in each image. For crack defects, the crack centerlines were manually traced and then dilated according to the observed crack width to generate pixel-level crack masks. For water leakage defects, polygonal boundaries were manually drawn along the visible edges of leakage stains. The annotation principle was to include only clearly visible defect areas and to avoid labeling shadows, stains, joints, or texture variations that could not be confidently identified as target defects. After the initial annotation, all masks were manually inspected and corrected by the research team to ensure annotation consistency and reduce subjective errors. The finalized pixel-wise masks were then exported as the ground truth data for model training and evaluation. To maintain consistency during the training process, all images and corresponding Ground Truth masks were resized to 512 × 512 pixels. Representative dataset samples are shown in Figs 1 and 2. Selected raw images data used in this study are provided in S1 Data.

**Fig 3:**
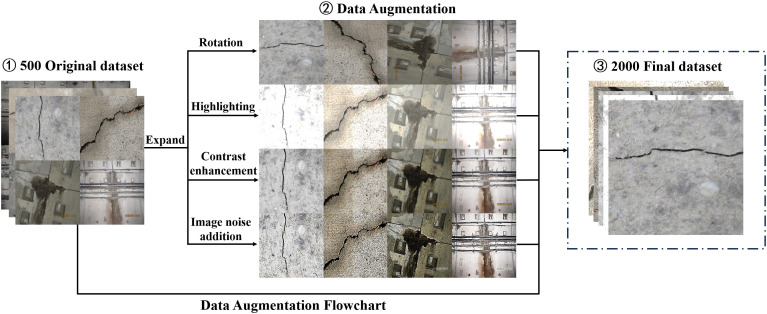
Data augmentation process. The composite figure was prepared by the authors based on model outputs generated from publicly available dataset images cited as Refs. [30,31] and selected author-provided raw images available in S1 Data.

To further enhance the model’s generalization and robustness, data augmentation was applied to both constructed datasets. Techniques such as rotation and brightness adjustment were employed, with the complete workflow detailed in Fig 3. This process expanded the original datasets, increasing the crack dataset to 800 images and the water leakage dataset to 1200 images, thereby effectively enhancing both the diversity and quantity of training data.

To mitigate the impact of class imbalance on model training, the dataset is randomly partitioned into training and validation sets in an 8:2 ratio. The training set is employed for updating and optimizing model weights, while the validation set is utilized to assess the model’s generalization performance on unseen samples.

### 2.2. Modeling

This study employs TransUNet as the backbone network for semantic segmentation, enabling precise segmentation of tunnel cracks and water leakage within images. TransUNet is a hybrid architecture integrating Convolutional Neural Networks (CNNs) with Vision Transformers (ViTs), possessing strong local feature extraction capabilities and long-range dependency modeling abilities, thereby facilitating effective segmentation of fine-grained structures.

The model architecture employs ResNet50V2 as its backbone network, a deep convolutional neural network responsible for extracting key low-level and high-level features from input images. Subsequently, these features are partitioned into non-overlapping image patches of 16 × 16 dimensions. These patches undergo linear transformation to embed them as vector sequences, serving as input to the Transformer. To enhance the model’s understanding of spatial structure, learnable positional embeddings are introduced to preserve the relative positional information between patches within the image. The embedded sequence is then fed into a Transformer module comprising twelve encoder layers. This structure models long-range dependencies and contextual information between features, thereby enhancing the model’s perception of complex structures and semantic boundaries. Building upon this foundation, the model incorporates decoder architecture. Through progressive upsampling and feature fusion, this recovers high-resolution images. The decoder consists of multiple convolutional layers that further refine the feature maps, ultimately producing precise segmentation results.

**Fig 4:**
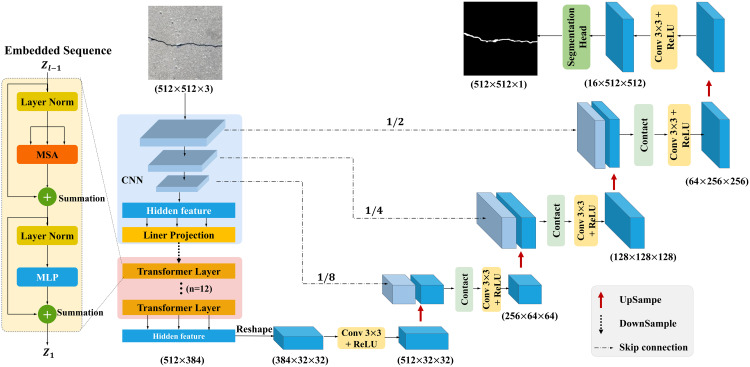
Architecture of the TransUNet model. This figure was prepared by the authors based on model outputs generated from selected raw images available in S1 Data.

In this study, the proposed TransUNet model was also compared with other high-performing models, including U-Net and DeepLabv3+. U-Net adopts an “encoder-decoder” architecture with skip connections, exhibiting weaker global context and multi-scale perception capabilities but lower computational demands, making it suitable for segmentation tasks with sharp edges and simple structures; DeepLabv3+ integrates the Atrous Spatial Pyramid Pooling (ASPP) module within its encoder-decoder framework, offering robust multi-scale information extraction capabilities with moderate computational overhead. It is well-suited for image segmentation in complex scenes with significant scale variations; TransUNet combines a Transformer encoder with a U-Net decoder, excelling in global modeling capabilities with moderate multiscale information processing. Its higher computational cost makes it suitable for high-precision segmentation applications requiring the fusion of global and local information. The network architecture of TransUNet is illustrated in Fig 4.

### 2.3. Evaluation metrics

To systematically evaluate the performance of various models in the task of semantic segmentation for tunnel defects, the study examines multiple dimensions including training convergence, comparative performance metrics, category-specific analysis, and engineering efficiency. Evaluation employs pixel-level metrics including Precision, Recall, F1-score, IoU, and overall Accuracy for quantitative assessment, supplemented by analysis of visualized prediction results. Concurrently, to demonstrate the models’ feasibility in engineering applications, the inference time and frame rate (FPS) of the three models were compared on a unified hardware platform to characterize the trade-off between accuracy and inference efficiency.

In the comprehensive evaluation of model performance and validity, five core metrics, including precision, recall, F1-score, IoU, and accuracy, are all grounded in the four fundamental components of the confusion matrix: true positives (TP), false positives (FP), true negatives (TN), and false negatives (FN). Precision measures the proportion of predicted positive samples that are genuinely positive, reflecting the model’s ability to minimize false positives. In tunnel crack and water leakage detection, high precision indicates that areas flagged as defects rarely misclassify background features, such as shadows or oil stains, as defects.

Recall, on the other hand, measures the proportion of actual positives that are correctly identified, indicating the model’s ability to avoid false negatives. For tunnel crack and water leakage detection, high recall ensures that even early or minor defects are detected, reducing operational safety risks. The F1-score represents the harmonic mean of Precision and Recall, offering a balanced measure of model performance, especially in class-imbalanced scenarios, such as medical image segmentation or small object detection. In tunnel images, where the foreground pixels represent less than 1% of the total image, the F1-score provides a fair assessment of the model’s ability to capture rare defect pixels.

The IoU quantifies the spatial overlap between predicted and ground-truth regions by calculating the ratio of their intersection to their union. As a critical metric for segmentation tasks, high IoU directly correlates with the accuracy of geometric measurements, such as crack length and water leakage area, which are essential for reliable maintenance decision-making. Accuracy provides an overall measure of correct predictions across all categories, offering a global perspective. In tunnel images, where the background area vastly exceeds the affected area, a model predicting the entire image as background might still achieve high accuracy, yet overlook cracks and water leakage. Therefore, joint evaluation with IoU is crucial. While Accuracy serves as a quick check for model bias toward background prediction, IoU ensures precise evaluation of the foreground defect areas. This combined approach prevents overestimation of model performance due to background dominance and ensures reliable detection of both overall structure and defect pixels.

The definitions and mathematical expressions for the aforementioned metrics are provided in Equations (1)–(5).


$$\begin{array}{c} \begin{array}{c} Precision\;=\;\frac{TP}{TP+FP} \end{array} \end{array}$$ (1)

$$\begin{array}{c} \begin{array}{c} Recall\;=\;\frac{TP}{TP+FN} \end{array} \end{array}$$ (2)

$$\begin{array}{c} \begin{array}{c} F\text{1}\;=\text{2}*\;\frac{Precision*Recall}{Precision+Recall} \end{array} \end{array}$$ (3)

$$\begin{array}{c} \begin{array}{c} IoU=\frac{TP}{\left(TP\;+FP+FN\right)} \end{array} \end{array}$$ (4)

$$\begin{array}{c} \begin{array}{c} Accuracy=\frac{TP+TN}{\left(TP+FP+TN+FN\right)} \end{array} \end{array}$$ (5)

### 2.4. Tunnel distress measurement algorithm

The two-dimensional characteristics of cracks primarily encompass their length and average width. For the crack mask image generated by the semantic segmentation algorithm, a skeletonization algorithm is first applied to extract the skeleton image of the crack, yielding a sequence of central line pixel coordinates. The total length *L* of the crack is calculated by summing the Euclidean distances between adjacent pixels along the skeleton:


$$\begin{array}{c} \begin{array}{c} L=\sum_{i=\text{1}}^{n-\text{1}}\sqrt{{\left(x_{i+\text{1}}-x_{i}\right)}^{\text{2}}+{\left(y_{i+\text{1}}-y_{i}\right)}^{\text{2}}} \end{array} \end{array}$$ (6)

In Equation (6), the crack skeleton is represented as an ordered sequence of pixel coordinates, denoted as ($x_{\text{1}},y_{\text{1}}$), ($x_{\text{2}},y_{\text{2}}$), …, ($x_{n},y_{n}$), where *n* is the total number of skeleton pixels. The Euclidean distance between each pair of adjacent skeleton points, ($x_{i},y_{i}$) and ($x_{i+\text{1}},y_{i+\text{1}}$), is calculated. The total crack length, measured in pixels, is then obtained by summing these distances over all consecutive point pairs from i=1 to n−1.

When calculating the average crack width, the average width may be determined by dividing the total cross-sectional area of the cracks by their combined length.


$$\begin{array}{c} \begin{array}{c} \overset{―}{\omega }=\frac{A}{L} \end{array} \end{array}$$ (7)

In a two-dimensional pit mask image, each pixel has a constant area of 1 pixel² in the image space. Therefore, the total pixel area 𝐴 of the crack is calculated by summing all foreground pixels in the mask. Here, *L* denotes the crack length obtained using Equation (6), and the average width $\overset{―}{\omega }$ is also expressed in pixels.

A calibration factor *k* (mm/pixel) can be obtained using a reference object with a known physical length placed in the same imaging plane as the tunnel distress, or through camera calibration under fixed acquisition conditions. Based on this factor, pixel-scale measurements can be converted into physical dimensions as follows: *length (mm)* = *pixel length* × *k*, *area (mm²)* = *pixel area* × *k*^2^. This conversion is applicable when the image has been geometrically calibrated or when the local imaging plane can be approximated as planar.

## 3. Model performance evaluation

To comprehensively evaluate the performance of the proposed method in tunnel crack and water leakage detection tasks, this study categorizes experiments into two distinct tasks: crack detection and leak detection. Each task undergoes independent training and validation. This experimental design not only facilitates targeted analysis of the model’s adaptability and feature extraction capabilities across different defect types, but also avoids feature conflicts and optimization instability arising from excessive variation in target features.

Tunnel cracks and water leakage, though both classified as surface defects in structural integrity, exhibit markedly distinct imaging characteristics. Cracks typically manifest slender, low-contrast linear features, demanding models with high edge sensitivity and the capacity to capture fine-grained structures. Water leakage, conversely, often appears as patchy regions with complex textures and indistinct boundaries, placing greater emphasis on models’ ability to model global context and maintain robustness to lighting variations. Training both types of defects concurrently within the same model may lead to imbalanced feature distributions, hindering the model’s ability to balance local fine-grained structures with global consistency during feature learning. This, in turn, compromises segmentation accuracy and convergence efficiency.

On this basis, the present study employs a dual-task independent training strategy. Under identical training frameworks and hyperparameter settings, separate training and testing are conducted for the crack and water leakage models respectively, thereby ensuring the comparability and specificity of the experimental results. The experimental hardware platform comprises an Intel® Xeon® CPU E5-2686v4@2.30GHz and an NVIDIA RTX A4000 GPU. The software stack includes Ubuntu 22.04, CUDA 12.1, cuDNN 8, and PyTorch 2.6.0. This study employed the following configuration for model training: a training cycle of 100 epochs, a batch size of 16, an initial learning rate of 1 × 10^−2^, and input image dimensions of 512 × 512 pixels. The author-generated code supporting model training, inference, performance evaluation, tunnel distress measurement, and statistical analysis is provided in S1 Code.

### 3.1. Training and convergence analysis for crack detection models

**Fig 5:**
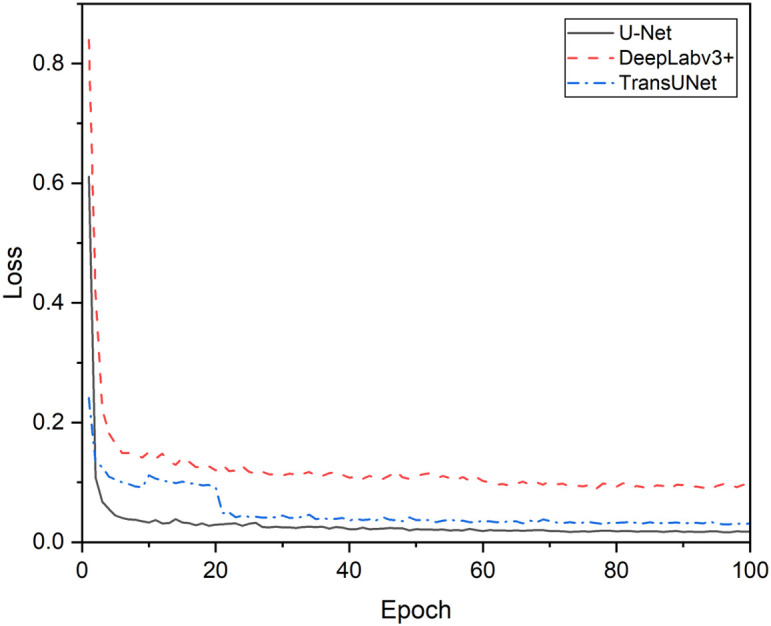
Training loss curves of three models for the crack detection task.

In the crack detection task, the training process is depicted in Fig 5. All three models, U-Net, DeepLabv3+, and TransUNet, entered the effective learning phase early on. Specifically, U-Net, DeepLabv3 + , and TransUNet achieved a cumulative reduction of 50% in total loss at epochs 3, 4, and 6, respectively, reaching a 95% reduction by epochs 8, 20, and 38. Following this, the loss curves stabilized into a low-amplitude oscillation phase within the convergence interval. Using a uniform criterion to define the plateau phase (based on a rolling average magnitude threshold), U-Net, DeepLabv3+, and TransUNet reached their plateau at epochs 43, 64, and 86, respectively. The corresponding smoothed minimum training losses were 0.017, 0.092, and 0.031.

Regarding optimization stability, TransUNet exhibited the lowest mean rolling standard deviation throughout the training process, approximately 0.006, followed by U-Net and DeepLabv3+, with values of 0.016 and 0.021, respectively. In the late stages of training (the final 20% of iterations), U-Net displayed the lowest standard deviation, around 0.00043, while TransUNet followed at approximately 0.00063, and DeepLabv3+ had a slightly higher value of 0.00103.

Overall, all three models rapidly established effective representations within the first 10 epochs. U-Net plateaued earlier and achieved the lowest training loss, while TransUNet exhibited smaller oscillations and greater optimization stability throughout the training process. DeepLabv3+ displayed convergence speed and overall performance that fell between the other two models.

As shown in Table 1, TransUNet achieved the highest performance on the test set, with an IoU of 71.57% and an accuracy of 98.38%. It also outperformed the other models in precision, achieving 82.42%. U-Net, on the other hand, had a slightly higher Recall of 81.81% and F1-score of 80.55% compared to TransUNet. DeepLabv3+ recorded relatively lower metrics across all evaluations. Overall, the results suggest that incorporating the long-range dependency modeling capabilities of Transformers enhances the spatial connectivity and boundary consistency of elongated, low-contrast seepage cracks. This improvement leads to better overall detection performance, with the most notable enhancement observed in the IoU metric.

**Table 1:** Performance comparison of the three models on the crack detection task.

Model	Precision	Recall	F1-score	IoU	Accuracy
**U-Net**	81.91%	81.81%	80.55%	71.13%	98.33%
**DeepLabv3+**	80.53%	78.10%	77.42%	67.51%	98.18%
**TransUNet**	82.42%	80.96%	80.49%	71.57%	98.38%

### 3.2. Training and convergence analysis for leak detection models

**Fig 6:**
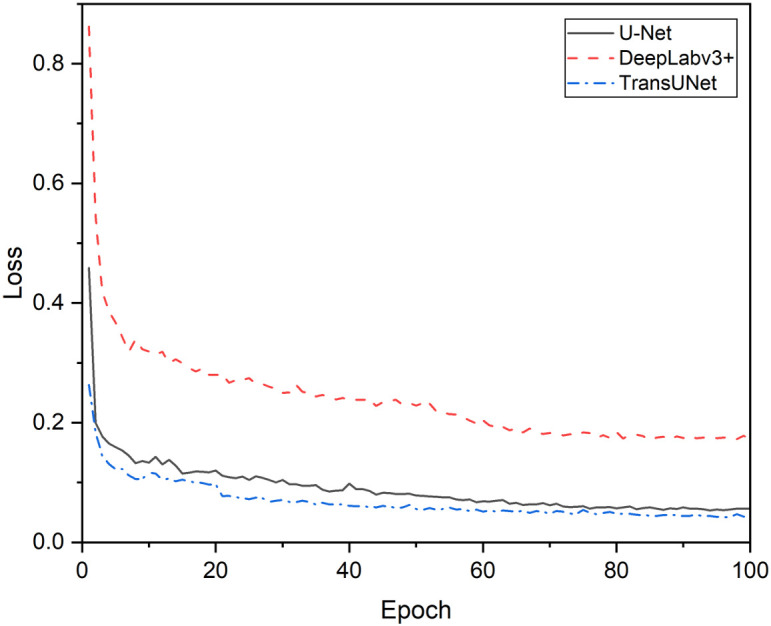
Training loss curves of three models for the water leakage detection task.

In the tunnel water leakage detection task, the training process is illustrated in Fig 6. All three models—U-Net, DeepLabv3+, and TransUNet—entered the effective learning phase early on. Specifically, U-Net, DeepLabv3+, and TransUNet achieved a cumulative 50% reduction in training loss at epochs 2, 3, and 4, respectively, reaching approximately 80% reduction at epochs 36, 85, and 58. Following this, the loss curves entered a low-amplitude oscillation phase within the convergence interval. The plateau phase, defined using a unified criterion (based on a rolling average gradient threshold), began at epochs 47, 85, and 28 for U-Net, DeepLabv3+, and TransUNet, respectively. The corresponding smoothed minimum training losses were approximately 0.054, 0.174, and 0.043.

In terms of optimization stability, TransUNet exhibited the lowest mean standard deviation throughout training, approximately 0.005, followed by U-Net and DeepLabv3+ with values of approximately 0.009 and 0.015, respectively. In the final 20% of training iterations, U-Net demonstrated the lowest standard deviation, approximately 0.0016. TransUNet followed with a value of approximately 0.0019, while DeepLabv3+ showed a slightly higher value of 0.0024.

Overall, all three models quickly established effective representations within the first 10 epochs. TransUNet reached plateau earlier, with smaller oscillations and more stable optimization throughout. U-Net achieved a comparably low training loss during the plateau phase, while DeepLabv3+ lagged behind both in convergence speed and overall performance.

As shown in Table 2, TransUNet achieved the best performance on the test set with a Precision of 91.51%, Recall of 70.42%, and Accuracy of 93.19%. Meanwhile, U-Net’s F1-score of 79.21% and IoU of 66.69% surpassed the other two models. DeepLabv3+ performs relatively poorly across various metrics. Overall, the results indicate that incorporating the long-range dependency modeling capabilities of Transformers enhance the detection rate of elongated, low-contrast leakage bands and improves overall discrimination performance. However, the model still falls slightly short of the more compact U-Net architecture in terms of region overlap (IoU) and overall balanced performance (F1 score). This suggests potential for further refinement in boundary refinement and small-object consistency in subsequent iterations.

**Table 2:** Performance comparison of the three models on the water leakage detection task.

Model	Precision	Recall	F1-score	IoU	Accuracy
**U-Net**	89.01%	67.50%	79.21%	66.69%	93.06%
**DeepLabv3+**	90.38%	66.48%	75.75%	62.60%	92.40%
**TransUNet**	91.51%	70.42%	76.78%	62.31%	93.19%

### 3.3. Model efficiency and engineering applicability

This study evaluated the inference efficiency and real-time performance of the three models on the test set, with results presented in Table 3. For the crack detection task, the DeepLabv3+ model exhibited the lowest average inference latency at 66 ms per image, demonstrating the highest processing speed. The U-Net model, with its relatively simpler architecture, recorded a latency of 89 ms per image. TransUNet, despite incorporating a Transformer encoding module which slightly increased latency, still maintained high inference efficiency. In the Water-leakage task, the inference speeds of all three models generally decreased. U-Net achieved an average latency of 172 ms per image, DeepLabv3+ reached 179 ms per image, and TransUNet recorded 199 ms per image.

**Table 3:** Inference efficiency for the three models.

Model	Crack		Water leakage	
	Inference Delay (ms/pic)	FPS	Inference Delay (ms/pic)	FPS
**U-Net**	89.00	11.13	172.00	5.81
**DeepLabv3+**	66.00	14.97	179.00	5.58
**TransUNet**	80.00	12.38	199.00	5.02

Overall, U-Net retains a slight edge in speed, while DeepLabv3+ exhibits the highest inference latency due to its greater network depth. TransUNet, however, maintains near-real-time inference speeds while preserving favorable accuracy, demonstrating an excellent balance between precision and efficiency.

Moreover, TransUNet achieves an average latency below 200 ms per image across both tasks, enabling millisecond-level inference. Coupled with its validated segmentation accuracy, it can be directly integrated into on-site inspection workflows. This eliminates the need for additional hardware upgrades while meeting the requirements for rapid screening of tunnel defects, demonstrating engineering feasibility.

### 3.4. Visualization and error analysis

**Fig 7:**
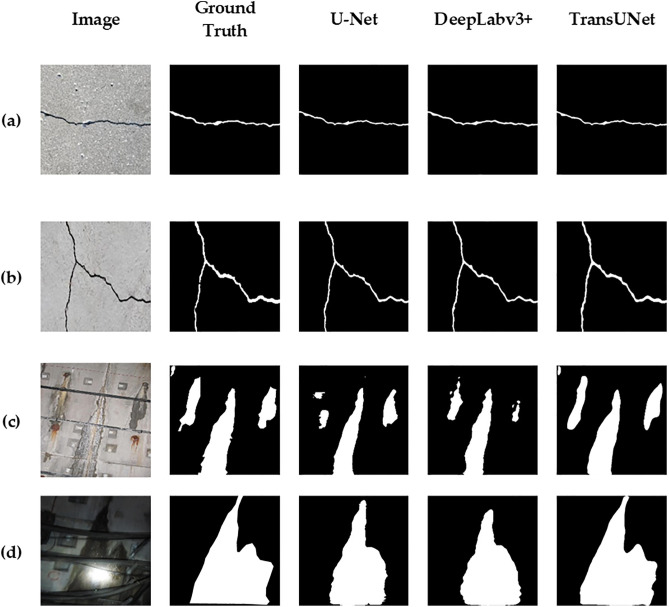
Comparison of segmentation results among the three models. This figure was prepared by the authors based on model outputs generated from selected raw images available in S1 Data.

To provide a more intuitive demonstration of the performance of each model in complex scenarios, this study selected representative samples from the test set for segmentation inference and conducted a visual comparative analysis of the output results, as shown in Fig 7. In crack detection, TransUNet effectively preserves crack connectivity, exhibiting fewer instances of discontinuity and false negatives. For water leakage detection, TransUNet accurately separates areas of reflective interference, producing smoother segmentation boundaries that align more closely with ground truth annotations. In contrast, U-Net frequently misses lesions in low-contrast regions, while DeepLabv3 + tends to exhibit blurred transitions at boundary adhesions. This qualitative outcome aligns with the quantitative metrics from the same test set, further validating TransUNet’s capability for global feature modeling and boundary delineation within complex contexts. Specific results are presented in Table 4.

**Table 4:** Comparison of segmentation performance among the three models.

Sample	Indicator	U-Net	DeepLabv3+	TransUNet
**(a)**	IoU	73.20%	76.71%	75.94%
	Precision	78.02%	86.26%	90.88%
	Recall	92.21%	87.38%	82.21%
	F1	84.52%	86.82%	86.33%
**(b)**	IoU	69.13%	70.60%	76.99%
	Precision	72.73%	75.40%	88.36%
	Recall	93.32%	91.72%	85.69%
	F1	81.75%	82.76%	87.00%
**(c)**	IoU	48.91%	50.22%	80.40%
	Precision	54.92%	54.73%	87.27%
	Recall	81.71%	85.90%	91.07%
	F1	65.69%	66.86%	89.14%
**(d)**	IoU	69.93%	71.38%	93.70%
	Precision	72.32%	73.12%	96.38%
	Recall	95.49%	96.77%	97.12%
	F1	82.31%	83.38%	96.75%

Building upon visual comparisons, this study conducted a geometric error analysis between model predictions and ground truth annotations to further evaluate the model’s predictive accuracy at the geometric scale. For the crack detection task, the relative errors in length and average width were calculated between predicted and annotated cracks. For the water leakage detection task, the relative error in area between predicted and annotated regions was computed to quantitatively characterize the model’s performance in shape preservation and scale consistency.

As shown in Table 5, all three methods achieved high accuracy in the quantitative characterization of cracks and water leakage, with TransUNet demonstrating the most balanced overall performance. Regarding crack geometric features, the relative errors in crack length for the three methods in Case (a) were 0.72% (U-Net), 0.09% (DeepLabv3+), and 0.48% (TransUNet), while in Case (b) they were 4.42%, 0.76%, and 1.21%. It can be observed that although DeepLabv3 + holds a slight advantage in length estimation, TransUNet exhibits a very similar error level, with both models accurately restoring the crack extension scale. Particularly in cases like (b), where cracks are longer and more complex in morphology, TransUNet maintains a low length error, demonstrating strong generalisation capabilities.

**Table 5:** Comparison of calculation results across different models.

Sample	Dimension	Indicator	U-Net	DeepLabv3+	TransUNet
**(a)**	**Length**	Ground truth (pixel)	196.24	196.24	196.24
		Calc. (pixel)	197.66	196.06	197.19
		Err. (%)	0.72	0.09	0.48
	**Width**	Ground truth (pixel)	2.78	2.78	2.78
		Calc. (pixel)	2.34	2.75	2.36
		Err. (%)	15.83	1.08	15.11
**(b)**	**Length**	Ground truth (pixel)	793.57	793.57	793.57
		Calc. (pixel)	828.61	799.61	783.96
		Err. (%)	4.42	0.76	1.21
	**Width**	Ground truth (pixel)	7.41	7.41	7.41
		Calc. (pixel)	5.62	6.13	7.77
		Err. (%)	24.16	17.27	4.86
**(c)**	**Area**	Ground truth (pixel)	6919	6919	6919
		Calc. (pixel)	4650	4406	6629
		Err. (%)	32.79	36.32	4.19
**(d)**	**Area**	Ground truth (pixel)	21499	21499	21499
		Calc. (pixel)	16406	16364	21534
		Err. (%)	23.69	23.88	0.16

Regarding crack width estimation, the relative errors for Case (a) were 15.83% (U-Net), 1.08% (DeepLabv3+), and 15.11% (TransUNet), while those for Case (b) were 24.16%, 17.27%, and 4.86%. TransUNet exhibited the lowest average width error in case (b), demonstrating greater sensitivity to minute width variations and thus proving more advantageous for the detailed characterisation of complex crack cross-sections.

When detecting water leakage areas, TransUNet shows a clear advantage. In Case (c), the relative errors in water leakage area were 32.79% for U-Net, 36.32% for DeepLabv3 + , and just 4.19% for TransUNet. In Case (d), the errors were 23.69% for U-Net, 23.88% for DeepLabv3 + , and only 0.16% for TransUNet. These results clearly show that TransUNet’s area estimates in both water leakage cases are much closer to the reference values, with errors significantly lower than those of the other two methods.

It’s clear that TransUNet not only performs exceptionally well in pixel-level segmentation for both crack and water leakage detection but also shows better control over geometric scale errors. This means the model accurately preserves the true size and shape of defects, providing a solid foundation for accurate defect assessment and structural safety evaluation.

## 4. Model validation and empirical analysis

### 4.1. Results analysis

This study collected inspection data from cracks and water leakage areas within the lining of a tunnel in Jiangxi Province. Under complex lighting conditions, high-resolution industrial cameras were employed to capture high-quality two-dimensional imagery, fully preserving the detailed characteristics of cracks and water leakage boundaries. This provides a robust foundation for subsequent precise quantitative analysis of crack length, width, and water leakage extent, thereby helping to mitigate systematic errors arising from environmental noise and reflections. The pixel-level measurement results, presented in Tables 6 and 7, demonstrate that the proposed method maintains average errors of less than 18% and 24% for crack length and width extraction, respectively. Moreover, the absolute deviation for most samples remains within the 5%–15% range, reflecting the algorithm’s repeatable and stable performance in skeleton path and edge localization. Regarding water leakage area identification, the overall mean error was controlled at 5.4%, with nine sample groups exhibiting pixel-level area discrepancies below 10%. This demonstrates the algorithm’s high-fidelity segmentation capability for identifying water stain boundaries.

**Table 6:** Calculation results for tunnel crack length and width.

Number	Tunnel crack length			Tunnel crack width		
	Ground truth (pixel)	Calc. (pixel)	Error (%)	Ground truth (pixel)	Calc. (pixel)	Error (%)
1	8436.42	7949.10	5.78	0.60	0.46	23.33
2	36787.33	33543.16	8.82	0.18	0.18	0.00
3	6144.31	5951.07	3.15	2.36	2.09	11.44
4	549.34	546.84	0.46	6.82	4.38	35.78
5	1110.62	949.23	14.53	4.78	3.89	18.62
6	26194.04	16752.73	36.04	0.44	0.60	36.36
7	1731.57	1384.86	20.02	5.46	5.45	0.18
8	4671.47	4549.87	2.60	2.15	1.79	16.74
9	2615.23	1664.00	36.37	3.15	4.59	45.71
10	1121.01	1657.51	47.86	7.62	3.77	50.52

**Table 7:** Calculation results for tunnel water leakage area.

Number	Tunnel water leakage area		
	Ground truth (pixel)	Calc. (pixel)	Error (%)
1	104136.00	104584.00	0.43
2	50557.00	48444.00	4.18
3	95304.00	102249.00	7.29
4	92525.00	86487.00	6.53
5	26206.00	25392.00	3.11
6	65594.00	64646.00	1.45
7	54556.00	43701.00	19.90
8	22306.00	20287.00	9.05
9	48820.00	47704.00	2.29
10	149693.00	150096.00	0.27

**Fig 8:**
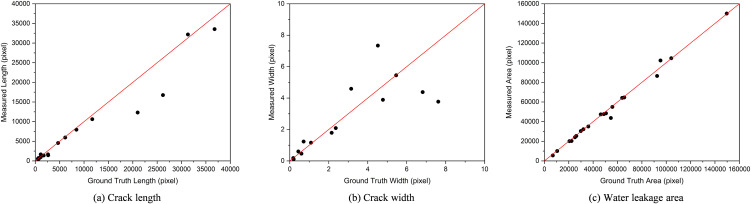
Validation of measurement accuracy. (a) Crack length measurement. (b) Crack width measurement. (c) Water leakage area measurement.

As shown in Fig 8, the measured values of tunnel crack length, width, and water leakage area all exhibit significant linear correlation with their true values, with R^2^ values of 0.98, 0.97, and 0.99, respectively. To further evaluate measurement accuracy, this study employs three metrics for quantitative analysis: root mean square error (RMSE), mean absolute percentage error (MAPE), and mean forecast error (MFE). Among these, MAPE is employed to evaluate forecast accuracy, while RMSE quantifies the deviation between observed and true values, exhibiting heightened sensitivity to outliers. Conversely, MFE reflects the concentration of forecast errors, its value being influenced by the mutual cancellation of positive and negative deviations.

For tunnel defect detection, the results show that the RMSE for crack length was 3089.97 pixels, with a MAPE of 16.25% and an MFE of 1419.54 pixels. For crack width, the RMSE was 5.65 pixels, the MAPE was 26.85%, and the MFE was 1.28 pixels. For water leakage area, the RMSE was 3322.68 pixels, the MAPE was 4.68%, and the MFE was 952.60 pixels. Although the RMSE and MAPE values indicate some degree of error between the predicted and actual values, the relatively small MFE values suggest that positive and negative errors tend to cancel each other out. This results in a more concentrated overall prediction bias. Consequently, the model’s measurement demonstrates good accuracy and consistency.

**Fig 9:**
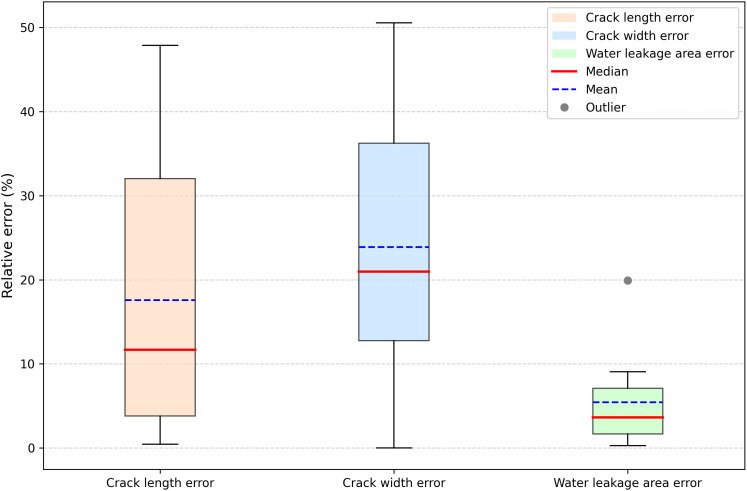
Boxplots of sample-wise relative errors for crack length, crack width, and water leakage area measurements.

Fig 9 presents boxplots of the sample-wise relative errors for crack length, crack width, and water leakage area measurements. For crack length estimation, the mean relative error is 17.56% with a standard deviation of 16.89%, while the median error is 11.68%. The interquartile range extends from 3.81% to 32.03%, indicating moderate variability among the test samples. Crack width estimation shows a relatively higher mean error of 23.87% and a median error of 20.98%, with an interquartile range from 12.76% to 36.22%. This suggests that crack width measurement is more sensitive to local segmentation deviations, especially for thin or irregular cracks. In contrast, water leakage area estimation achieves a much lower mean relative error of 5.45% and a median error of 3.64%, with an interquartile range from 1.66% to 7.10%, demonstrating higher accuracy and stability. Although one outlier is observed in the water leakage area error distribution, the overall error level remains relatively low. These results indicate that area-based measurement is the most stable, followed by crack length estimation, whereas crack width estimation exhibits the largest deviations and remains the most challenging measurement task.

### 4.2. Measurement limitations and engineering applicability

Although the proposed framework demonstrates the feasibility of segmentation-based tunnel distress measurement, several limitations should be noted regarding its engineering application. First, the geometric quantities in this study are reported in pixel units because the primary objective was to evaluate the consistency and feasibility of the proposed measurement framework. For practical engineering applications, pixel-scale measurements can be converted into physical dimensions by introducing a reference object, scale marker, or camera calibration procedure during image acquisition. In future field deployment, geometric calibration and physical-scale conversion will be incorporated to further improve the direct applicability of the proposed method for tunnel distress assessment.

Second, the crack width estimation method employed in this study calculates the average width as the ratio of the crack area to its skeleton length. This approach provides a simple and intuitive indicator of the overall crack width, but it may have limited applicability for cracks with highly non-uniform widths, branching structures, or irregular boundaries. In particular, the average width may fail to capture local width variations and critical maximum widths, which are often more relevant to structural safety assessment. Future research will address this limitation by integrating local normal-direction width measurement and sub-pixel boundary extraction techniques to obtain more accurate and reliable crack width measurements.

## 5. Conclusions

The proposed TransUNet-based tunnel lining defect segmentation framework significantly enhances the delineation of slender cracks and ambiguous water leakage boundaries under complex lighting and textural interference conditions. This is achieved by integrating CNN’s local texture representation with Transformer’s global dependency modeling. Compared with U‑Net and DeepLabv3+, the proposed TransUNet framework demonstrates advantages in precision, boundary preservation, and crack detection. While U‑Net achieves higher IoU for water leakage, TransUNet provides a more balanced performance across both defect types, together with high inference efficiency, making it a practical choice for tunnel inspection. Beyond technical performance, this research offers a critical perception layer for sustainable transportation management. By enabling the transition from reactive to proactive maintenance, the proposed framework enhances the structural resilience of tunnel lifelines and minimizes the long-term environmental and economic costs associated with infrastructure degradation. Comprehensive experimental results indicate that incorporating a self-attention mechanism is a key approach to enhancing the robustness of tunnel defect segmentation. The key conclusions are as follows:

(1) Segmentation Performance: TransUNet achieves strong performance on both crack and water leakage detection. For cracks, it attains the highest IoU of 71.57% and an accuracy of 98.38%. For water leakage, it yields the highest precision of 91.51%, effectively reducing false positives, whereas U‑Net shows better region overlap with an IoU of 66.69%. Given the engineering priority of minimizing false alarms, TransUNet offers a compelling advantage.
(2) Superior Geometric Consistency: The method demonstrated superior geometric consistency compared to baseline models, making it suitable for engineering quantification. Mask-based geometric evaluations revealed a relative error of 0.48% for crack length and 4.19% for water leakage area, confirming that the pixel-level segmentation results are reliably mapped to engineering-relevant quantitative metrics such as length and area.
(3) Efficiency and Practical Applicability: Under consistent hardware conditions, the average inference latency was approximately 80 ms per image (12.38 FPS) for crack detection and 199 ms per image (5.02 FPS) for water leakage detection. These results meet the performance requirements for both offline inspection and near-real-time detection, while maintaining high accuracy.

Due to limitations in dataset diversity and hardware resources, TransUNet still faces challenges in fine-grained crack width estimation, generalization under extreme lighting conditions, and power efficiency on embedded devices. Future research will focus on expanding the dataset under more diverse tunnel environments, incorporating geometric calibration and physical-scale conversion, and improving crack width measurement through local normal-direction width estimation and sub-pixel boundary extraction. In addition, lightweight model design and edge-computing integration will be further explored to support large-scale deployment in real-time structural health monitoring systems, thereby enhancing the inspection efficiency and operational safety of critical tunnel infrastructure.

## Supporting information

S1 Data This file includes selected raw images data used in the manuscript. (ZIP)

S1 Code This file includes the code for model training, inference, evaluation, tunnel distress measurement, and statistical analysis. (ZIP)
