# Supplementary material for: Semantic segmentation and quantitative analysis of tunnel cracks and water leakage using a TransUNet framework
Source: PLoS One. 2026 Aug 11;21(8):e0349175. doi: 10.1371/journal.pone.0349175 (PMC13460598; doi:10.1371/journal.pone.0349175)
Supplement: Supplementary file 1 [file pone.0349175.s003.pdf]

Manuscript ID: PONE-D-26-20664

Title: Semantic Segmentation and Quantitative Analysis of Tunnel Cracks and Water Leakage Using a TransUNet Framework

Corresponding Author: Qifeng Yu (Email: qfyu@shmtu.edu.cn)

Date: July 4, 2026

Dear Academic Editor and Reviewers,

Thank you for your thoughtful and constructive comments on our manuscript. We have carefully addressed all the points raised by the journal and the reviewers. Below we provide a point-by-point response. All changes in the revised manuscript have been marked in red font.

JOURNAL REQUIREMENTS:

1. *Please ensure that your manuscript meets PLOS ONE's style requirements, including those for file naming.*

Response: The manuscript has been revised to meet PLOS ONE’s style requirements, including double-spacing, continuous line numbers, and proper file naming.

2. *In the online submission form, you indicated that the data presented in this study is available on request from the corresponding author. The data is not publicly available due to the privacy restrictions of the collaborating organization and safety regulations concerning infrastructure data.*

Response: We thank the editorial office for pointing out the PLOS data availability requirement. We have revised the Data Availability Statement accordingly. The previous statement indicating that the data was available upon request from the

corresponding author has been replaced.

The primary image data used in this study were derived from the publicly available datasets cited as Refs. [30,31] in the manuscript. To support reproducibility, selected raw images used in this study have been provided as S1 Data. The supporting file contains the data underlying the quantitative results, tables, and figures reported in the manuscript. Additional quantitative results are reported in the tables and figures of the revised manuscript.

3. *Thank you for stating the following financial disclosure: This research was funded by the Science and Technology Program of Guangxi, China (No. 2023AB08158). Please state what role the funders took in the study. If the funders had no role, please state: "The funders had no role in study design, data collection and analysis, decision to publish, or preparation of the manuscript." If this statement is not correct you must amend it as needed. Please include this amended Role of Funder statement in your cover letter; we will change the online submission form on your behalf.*

Response: We thank the editorial office for the clarification. We have amended the Funding Statement and added the required Role of Funder statement in the cover letter. The revised statement is as follows:

Funding Statement: This research was funded by the Science and Technology Program of Guangxi, China (No. 2023AB08158). The funder had no role in study design, data collection and analysis, decision to publish, or preparation of the manuscript.

4. *We note that Figure(s) 1, 2, 3, 4, 7 in your submission contain copyrighted images. All PLOS content is published under the Creative Commons Attribution License (CC BY 4.0), which means that the manuscript, images, and Supporting Information files will be freely available online, and any third party is permitted to access, download, copy, distribute, and use these materials in any way, even commercially, with proper attribution.*

Response: We thank the editorial office for pointing out the copyright requirements for Figures 1, 2, 3, 4, and 7. We have carefully reviewed the image sources used in these figures.

Some representative images in the figures were obtained from the publicly available datasets cited as Refs. [30,31] in the manuscript. These datasets are distributed under licenses compatible with publication under the Creative Commons Attribution License (CC BY 4.0). In addition, selected author-provided raw images used in the figures have been provided in S1 Data. The composite figures, model framework diagrams, experimental result visualizations, and statistical plots were prepared by the authors.

To avoid ambiguity, we have revised the relevant figure captions to clearly indicate the image sources and to distinguish publicly available dataset images from author-provided images. Therefore, no additional copyright permission is required for these figures to be published under the CC BY 4.0 license.

The revised figure captions:

Fig 1. Examples of tunnel crack dataset images and corresponding masks (top: original images; bottom: ground-truth images). The composite figure was prepared by the authors using images from the publicly available dataset reported in Ref. [30] and selected author-provided raw images available in S1 Data. The publicly available dataset images are distributed under a license compatible with publication under CC BY 4.0.

Fig 2. Examples of tunnel water leakage dataset images and corresponding masks (top: original images; bottom: ground-truth images). The composite figure was prepared by the authors using images from the publicly available dataset reported in Ref. [31] and selected author-provided raw images available in S1 Data. The publicly available dataset images are distributed under a license compatible with publication under CC BY 4.0.

Fig 3. Data augmentation process. The composite figure was prepared by the authors based on model outputs generated from publicly available dataset images cited as Refs. [30,31] and selected author-provided raw images available in S1 Data.

Fig 4. Architecture of the TransUNet model. This figure was prepared by the authors based on model outputs generated from selected raw images available in S1 Data.

Fig 7. Comparison of segmentation results among the three models. This figure was prepared by the authors based on model outputs generated from selected raw images available in S1 Data.

5. *Please note that PLOS One has specific guidelines on code sharing for submissions in which author-generated code underpins the findings in the manuscript. In these cases, we expect all author-generated code to be made available without restrictions upon publication of the work.*

Response: We thank the editorial office for reminding us of the PLOS ONE code sharing policy. To comply with this requirement, we have submitted the author-generated code as a Supporting Information file, "S1 Code," together with the revised manuscript. The submitted code includes scripts for model training, inference, evaluation, tunnel distress measurement, and statistical visualization.

6. *Please provide a complete Data Availability Statement in the submission form, ensuring you include all necessary access information or a reason for why you are unable to make your data freely accessible. If your research concerns only data provided within your submission, please write "All data are in the manuscript and/or supporting information files" as your Data Availability Statement.*

Response: We thank the editorial office for the reminder. We have revised the Data Availability Statement in the submission form to provide complete access information for the dataset. The revised Data Availability Statement is as follows:

Data Availability Statement: The primary image dataset used in this study was derived from the publicly available Tunnel Crack Dataset reported in Ref. [30, 31]. To support reproducibility, selected raw images used in this study have been provided as S1 Data. The supporting file contains the data underlying the quantitative results, tables, and figures reported in the manuscript.

7. *Thank you for stating the following in the Acknowledgments Section of your manuscript: This research was supported by the Royal Academy of Engineering (RAEng) through a research fellowship awarded to N. V.*

We note that you have provided funding information that is not currently declared in your Funding Statement. However, funding information should not appear in the Acknowledgments section or other areas of your manuscript. We will only publish funding information present in the Funding Statement section of the online submission form.

Please remove any funding-related text from the manuscript and let us know how you would like to update your Funding Statement. Currently, your Funding Statement reads as follows: Royal Academy of Engineering (RAEng) Research Fellowship to NV

Please include your amended statements within your cover letter; we will change the online submission form on your behalf.

Response: We thank the editorial office for pointing this out. We have carefully checked the manuscript and confirm that the statement regarding the Royal Academy of Engineering (RAEng) Research Fellowship awarded to N. V. was included in error. This funding source is not related to our manuscript, and our study was not supported by the Royal Academy of Engineering. We have removed the Acknowledgments section and any funding-related text from the manuscript. The Funding Statement should be updated as follows:

Funding Statement: This research was funded by the Science and Technology Program of Guangxi, China (No. 2023AB08158). The funders had no role in study

design, data collection and analysis, decision to publish, or preparation of the manuscript.

8. *Please update your submission to use the PLOS LaTeX template.*

Response: We thank the editorial office for the reminder regarding the PLOS LaTeX template. Our manuscript was prepared and submitted as a Word document rather than a LaTeX manuscript. Therefore, the LaTeX template requirement is not applicable to this submission. We have instead revised the manuscript according to the PLOS ONE formatting and style requirements for Word submissions.

9. *We note that Figure(s) 1-9 in your submission contain copyrighted images. All PLOS content is published under the Creative Commons Attribution License (CC BY 4.0), which means that the manuscript, images, and Supporting Information files will be freely available online, and any third party is permitted to access, download, copy, distribute, and use these materials in any way, even commercially, with proper attribution.*

Response: We thank the editorial office for pointing out the copyright requirements for Figures 1, 2, 3, 4, and 7. We have carefully reviewed the image sources used in these figures.

Some representative images in the figures were obtained from the publicly available datasets cited as Refs. [30,31] in the manuscript. These datasets are distributed under licenses compatible with publication under the Creative Commons Attribution License (CC BY 4.0). In addition, selected author-provided raw images used in the figures have been provided in S1 Data. The composite figures, model framework diagrams, experimental result visualizations, and statistical plots were prepared by the authors.

To avoid ambiguity, we have revised the relevant figure captions to clearly indicate the image sources and to distinguish publicly available dataset images from author-provided images. Therefore, no additional copyright permission is required for these

figures to be published under the CC BY 4.0 license.

The revised figure captions:

Fig 1. Examples of tunnel crack dataset images and corresponding masks (top: original images; bottom: ground-truth images). The composite figure was prepared by the authors using images from the publicly available dataset reported in Ref. [30] and selected author-provided raw images available in S1 Data. The publicly available dataset images are distributed under a license compatible with publication under CC BY 4.0.

Fig 2. Examples of tunnel water leakage dataset images and corresponding masks (top: original images; bottom: ground-truth images). The composite figure was prepared by the authors using images from the publicly available dataset reported in Ref. [31] and selected author-provided raw images available in S1 Data. The publicly available dataset images are distributed under a license compatible with publication under CC BY 4.0.

Fig 3. Data augmentation process. The composite figure was prepared by the authors based on model outputs generated from publicly available dataset images cited as Refs. [30,31] and selected author-provided raw images available in S1 Data.

Fig 4. Architecture of the TransUNet model. This figure was prepared by the authors based on model outputs generated from selected raw images available in S1 Data.

Fig 7. Comparison of segmentation results among the three models. This figure was prepared by the authors based on model outputs generated from selected raw images available in S1 Data.

All other figures were generated from our own experimental workflow, model results, statistical analyses, or author-prepared schematic illustrations, and were not reproduced, adapted, or reprinted from any previously published or third-party copyrighted sources.

10. *Please include captions for your Supporting Information files at the end of your manuscript, and update any in-text citations to match accordingly.*

Response: We thank the editorial office for the reminder. We have added captions for all Supporting Information files at the end of the revised manuscript. The in-text citations to the Supporting Information files have also been checked and updated to ensure consistency with the submitted files.

Supporting Information:

S1 Data. This file includes selected raw images data used in the manuscript.

S1 Code. This file includes the code for model training, inference, evaluation, tunnel distress measurement, and statistical analysis.

11. *If the reviewer comments include a recommendation to cite specific previously published works, please review and evaluate these publications to determine whether they are relevant and should be cited. There is no requirement to cite these works unless the editor has indicated otherwise.*

Response: We thank the editorial office for the clarification. The reviewer comments did not require the citation of specific previously published works. We have nevertheless reviewed the relevant literature during revision and updated the references where appropriate.

RESPONSES TO REVIEWER 1

We sincerely appreciate the reviewer’s careful and thorough evaluation of our manuscript. Your insightful comments were extremely valuable in helping us identify and correct several numerical inconsistencies, factual inaccuracies, and language issues, thereby substantially improving the rigor, clarity, and overall quality of the manuscript. Detailed responses to each comment are provided below.

Comment 1: The manuscript contains several terminology inaccuracies as well as inconsistencies between British and American English usage. The authors are encouraged to carefully proofread the manuscript and unify the writing style throughout. For example:

a) On Page 7, Line 157, “Attenuated Spatial Pyramid Pooling (ASPP)” is incorrect and should be revised to “Atrous Spatial Pyramid Pooling (ASPP)”;

b) Terms such as “lesion,” “pathologies,” and “disease types,” which are more commonly used in medical imaging literature, appear throughout the manuscript and may result from direct translation. It is recommended to replace them with terminology more appropriate for civil engineering contexts, such as “defect types,” “distress categories,” or “tunnel defects”;

c) British and American English spellings are mixed in the manuscript, such as “modelling/modeling” and “whilst/while.” A consistent language style should be adopted;

d) In Fig. 1 and Fig. 2, the term “masked images” is not sufficiently precise and is recommended to be replaced with “ground-truth masks” or “annotation masks.”

Authors’ Response:

We thank you for carefully identifying these terminology and language issues. We have systematically revised the manuscript as follows:

(a) In Section 2.2, we have corrected "Attenuated Spatial Pyramid Pooling (ASPP)" to "Atrous Spatial Pyramid Pooling (ASPP)".

(b) Throughout the manuscript, we have replaced medical terminology with civil engineering terms:

- In the Introduction, "lesion characteristics" have been changed to "defect characteristics".
- In Section 2.3, "lesion pixels" has been changed to "defect pixels".

- In Section 3, "disease types" has been changed to "defect types", and "pathologies" has been changed to "defects".

(c) The manuscript has been unified using American English: "whilst" has been replaced with "while" (e.g., in Section 2.1 on data augmentation), and "modelling" has been changed to "modeling" (e.g., in the title of Section 2.2).

(d) In the captions of Figures 1 and 2, "masked images" has been replaced with "ground-truth masks".

Comment 2: Although the literature review is generally comprehensive, it remains largely focused on conventional CNN-based approaches, while the discussion of recent Transformer-based methods for infrastructure defect detection is relatively limited. It is recommended that the authors further incorporate recent studies, such as: Applications of SegFormer in infrastructure defect detection; Fine-grained crack segmentation models such as CrackFormer and CrackSegFormer; Research related to lightweight Transformer architectures and boundary enhancement strategies.

Authors' Response:

Thank you for the suggestion to incorporate recent Transformer-based studies. We have expanded the literature review in the Introduction; please refer to lines 94-100 of the revised manuscript. The specific modifications are as follows:

"Recent studies have further advanced Transformer-based crack segmentation. For instance, CrackFormer-II embeds novel Transformer encoders into a SegNet-like architecture, achieving higher accuracy with substantially fewer computational resources [26]. LiteCrackSeg, a lightweight hybrid CNN–Transformer model, requires only 2.72 million parameters and achieves an inference speed of 56 frames per second, making it suitable for edge deployment [27]. Along this line of research, TransUNet has shown advantages over traditional CNN models in multi-scale feature representation, structural integrity restoration, and small-object detection, thereby offering improved accuracy and robustness for crack segmentation tasks."

The added related references are as follows:

26. Liu H, Yang J, Miao X, Mertz C, Kong H. CrackFormer network for pavement crack segmentation. IEEE Trans Intell Transp Syst 2023, 24, 9240-9252. https://doi.org/10.1109/TITS.2023.3266776

27. Gobena KA, Rakib MYK, Tesema FB, Asafa GF, Ren S. LiteCrackSeg: A lightweight hybrid CNN–transformer for efficient crack segmentation. PLOS ONE 2026, 21, e0347765. https://doi.org/10.1371/journal.pone.0347765

Comment 3: In the water leakage detection task, U-Net achieves better F1-score and IoU values than TransUNet. However, the manuscript repeatedly emphasizes that TransUNet demonstrates the best overall performance. It is recommended that the authors present the experimental findings in a more objective and balanced manner. For example: Emphasize the advantages of TransUNet in terms of Precision and boundary integrity; At the same time, acknowledge that U-Net demonstrates superior region overlap and overall balanced performance. In addition, overly absolute expressions such as “significantly outperforms” and “best overall performance” should be used more cautiously to improve the credibility and rigor of the discussion.

Authors’ Response:

We thank the reviewer for pointing out the need for a more objective and balanced presentation. We have revised the discussion in Section 3.2 and the Conclusions accordingly; please refer to lines 23-26 (in Abstract), lines 445-448 and lines 454-457 (in Conclusions) of the revised manuscript. The specific modifications are as follows:

“In terms of precision, boundary preservation, and geometric consistency, the proposed method shows clear advantages over the comparison models, while U-Net exhibits stronger region overlap for water leakage detection. Overall, the method meets the requirements of offline inspection and near-real-time applications.” Please refer to lines 23-26.

“Compared with U-Net and DeepLabv3+, the proposed TransUNet framework demonstrates advantages in precision, boundary preservation, and crack detection. While

U-Net achieves higher IoU for water leakage, TransUNet provides a more balanced performance across both defect types, together with high inference efficiency, making it a practical choice for tunnel inspection." Please refer to lines 445-448.

"Segmentation Performance: TransUNet achieves strong performance on both crack and water leakage detection. For cracks, it attains the highest IoU of 71.57% and an accuracy of 98.38%. For water leakage, it yields the highest precision of 91.51%, effectively reducing false positives, whereas U-Net shows better region overlap with an IoU of 66.69%. Given the engineering priority of minimizing false alarms, TransUNet offers a compelling advantage." Please refer to lines 454-457.

Comment 4: The description of the dataset annotation procedure is relatively brief. It is recommended that the authors provide additional details regarding: Annotation tools and annotation workflow; Annotator information and annotation principles; Whether manual verification and correction were performed. Furthermore, the acquisition process of the ground truth data should be clarified.

Authors' Response:

We thank the reviewer for the helpful suggestion regarding the need for a more detailed description of the dataset annotation procedure. In response, we have added a more comprehensive explanation in Section 2.1 of the revised manuscript. Please refer to Lines 126-137 for the corresponding revisions. The specific modifications are as follows:

"Among these images, approximately 70% were obtained from publicly available datasets [30,31], while the remaining 30% were collected from an in-service tunnel. Pixel-wise annotations were generated using LabelMe and Roboflow Annotate following a standardized annotation workflow. Specifically, trained annotators first identified the visible defect regions in each image. For crack defects, the crack centerlines were manually traced and then dilated according to the observed crack width to generate pixel-level crack masks. For water leakage defects, polygonal boundaries were manually drawn along the visible edges of leakage stains. The annotation principle was to include only clearly visible defect areas and to avoid labeling

shadows, stains, joints, or texture variations that could not be confidently identified as target defects. After the initial annotation, all masks were manually inspected and corrected by the research team to ensure annotation consistency and reduce subjective errors. The finalized pixel-wise masks were then exported as the ground truth data for model training and evaluation. To maintain consistency during the training process, all images and corresponding Ground Truth masks were resized to 512 × 512 pixels."

Comment 5: In the crack length calculation formula around Line 196, only the mathematical expression is currently provided, while the physical meanings of the variables and symbols are not sufficiently explained. It is recommended that the authors provide additional clarification regarding the definitions of the variables and the corresponding calculation process to improve readability and completeness.

Authors' Response:

We sincerely thank the reviewer for highlighting the need for a more detailed explanation. Accordingly, we have supplemented Section 2.4, "Tunnel Distress Measurement Algorithm," with additional clarification. Please refer to lines 225–228 and lines 232-234 of the revised manuscript for the corresponding revisions. The specific modifications are as follows:

"In Equation (6), the crack skeleton is represented as an ordered sequence of pixel coordinates, denoted as (x_1, y_1), (x_2, y_2), … , (x_n, y_n), where n is the total number of skeleton pixels. The Euclidean distance between each pair of adjacent skeleton points, (x_i, y_i) and (x_{i+1}, y_{i+1}) , is calculated. The total crack length L , measured in pixels, is then obtained by summing these distances over all consecutive point pairs from $i = 1$ to $n - 1$."

"In a two-dimensional pit mask image, each pixel has a constant area of 1 pixel² in the image space. Therefore, the total pixel area A of the crack is calculated by summing all foreground pixels in the mask. Here, L denotes the crack length obtained using Equation (6), and the average width $\overline{\omega}$ is also expressed in pixels."

RESPONSES TO REVIEWER 2

We are very grateful to you for the insightful and constructive suggestions, including the valuable references and recommendations on section structure, which have significantly strengthened our manuscript.

Comment 1: The manuscript mentions that both public datasets and engineering field images were adopted; however, important information regarding the dataset composition remains insufficiently described. In particular, the proportions of different data sources, annotation procedures, and dataset construction details are not clearly presented. Additional information regarding dataset preparation and annotation is needed to improve the reliability and reproducibility of the study.

Authors' Response:

We thank the reviewer for emphasizing the importance of dataset composition and reproducibility. We have supplemented Section 2.1 with the following information; please refer to lines 126-137 of the revised manuscript. The specific modifications are as follows:

"Among these images, approximately 70% were obtained from publicly available datasets [30,31], while the remaining 30% were collected from an in-service tunnel. Pixel-wise annotations were generated using LabelMe and Roboflow Annotate following a standardized annotation workflow. Specifically, trained annotators first identified the visible defect regions in each image. For crack defects, the crack centerlines were manually traced and then dilated according to the observed crack width to generate pixel-level crack masks. For water leakage defects, polygonal boundaries were manually drawn along the visible edges of leakage stains. The annotation principle was to include only clearly visible defect areas and to avoid labeling shadows, stains, joints, or texture variations that could not be confidently identified as target defects. After the initial annotation, all masks were manually inspected and corrected by the research team to ensure annotation consistency and reduce subjective errors. The finalized pixel-wise masks were then exported as the ground truth data for model training and evaluation. To maintain consistency during the training process, all images and corresponding Ground Truth masks were resized to 512 × 512 pixels."

Comment 2: The experimental evaluation is mainly based on metrics such as Precision, Recall, F1-score, and IoU. While these indicators are commonly used in semantic segmentation tasks, the statistical analysis of the experimental results is still relatively limited. It would be beneficial to further provide: mean and standard deviation (std) obtained from repeated experiments; error distribution analysis; boxplots or error distribution visualizations.

Authors' Response:

We thank the reviewer for recommending additional statistical analysis. Due to computational constraints, repeated training runs were not performed in this study. However, we have supplemented the evaluation with an error distribution analysis based on the ten field-test samples, as reported in Tables 6 and 7. Specifically, a boxplot visualization (Fig. 9) and corresponding statistical summaries have been added to Section 4. Please refer to Lines 412–425 of the revised manuscript for the corresponding revisions. The specific modifications are as follows:

"Fig. 9 presents boxplots of the sample-wise relative errors for crack length, crack width, and water leak-age area measurements. For crack length estimation, the mean relative error is 17.56% with a standard deviation of 16.89%, while the median error is 11.68%. The interquartile range extends from 3.81% to 32.03%, indicating moderate variability among the test samples. Crack width estimation shows a relatively higher mean error of 23.87% and a median error of 20.98%, with an interquartile range from 12.76% to 36.22%. This suggests that crack width measurement is more sensitive to local segmentation deviations, especially for thin or irregular cracks. In contrast, water leakage area estimation achieves a much lower mean relative error of 5.45% and a median error of 3.64%, with an interquartile range from 1.66% to 7.10%, demonstrating higher accuracy and stability. Although one outlier is observed in the water leakage area error distribution, the overall error level remains relatively low. These results indicate that area-based measurement is the most stable, followed by crack length estimation, whereas crack width estimation exhibits the largest deviations and remains the most challenging measurement task."

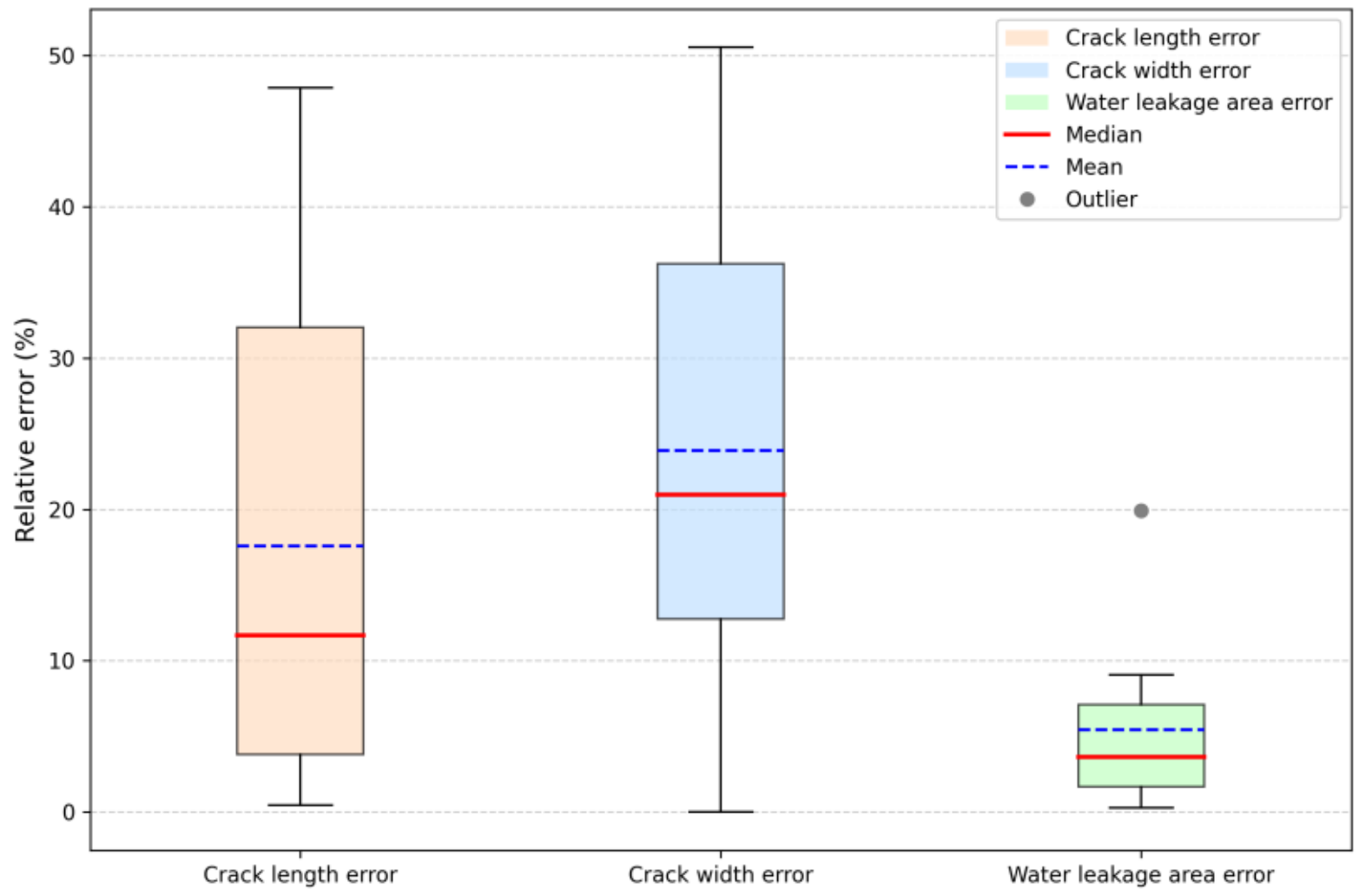

Fig 9. Boxplots of sample-wise relative errors for crack length, crack width, and water leakage area measurements.

Comment 3: Currently, crack length, crack width, and water leakage area are all quantified in pixel units, while the correspondence between image-space measurements and real-world engineering dimensions has not been sufficiently explained. Further clarification regarding the relationship between pixel-scale measurements and actual physical dimensions is recommended to strengthen the engineering applicability of the proposed framework.

Authors' Response:

We thank the reviewer for raising this important issue regarding the engineering applicability of the proposed measurement framework. We agree that the relationship between pixel-scale measurements and real-world physical dimensions should be further clarified. In response, we have added two clarifications in the revised manuscript.

First, at the end of Section 2.4, "Tunnel Distress Measurement Algorithm," we have supplemented the conversion relationship between image-space measurements and physical dimensions. Please refer to Lines 235-239 of the revised manuscript. The specific addition is as follows:

"*A calibration factor* k *(mm/pixel) can be obtained using a reference object with a known physical length placed in the same imaging plane as the tunnel distress, or through camera calibration under fixed acquisition conditions. Based on this factor, pixel-scale measurements can be converted into physical dimensions as follows: length (mm) = pixel length* $\times$ k*, area (mm²) = pixel area* $\times$ k^2*. This conversion is applicable when the image has been geometrically calibrated or when the local imaging plane can be approximated as planar.*"

Second, in Section 4.2, we have explicitly acknowledged the current limitation of reporting geometric quantities in pixels and clarified how physical-scale measurements can be incorporated in future field applications. Please refer to Lines 427-433 of the revised manuscript. The specific addition is as follows:

"*Although the proposed framework demonstrates the feasibility of segmentation-based tunnel distress measurement, several limitations should be noted regarding its engineering application. First, the geometric quantities in this study are reported in pixel units because the primary objective was to evaluate the consistency and feasibility of the proposed measurement framework. For practical engineering applications, pixel-scale measurements can be converted into physical dimensions by introducing a reference object, scale marker, or camera calibration procedure during image acquisition. In future field deployment, geometric calibration and physical-scale conversion will be incorporated to further improve the direct applicability of the proposed method for tunnel distress assessment.*"

Third, in Section 5, we have also updated the limitation of reporting geometric quantities in pixels. Please refer to Lines 466-472 of the revised manuscript. The specific addition is as follows:

"Due to limitations in dataset diversity and hardware resources, TransUNet still faces challenges in fi-ne-grained crack width estimation, generalization under extreme lighting conditions, and power efficiency on embedded devices. Future research will focus on expanding the dataset under more diverse tunnel environments, incorporating geometric calibration and physical-scale conversion, and improving crack width measurement through local normal-direction width estimation and sub-pixel boundary extraction. In addition, lightweight model design and edge-computing integration will be further explored to support large-scale deployment in real-time structural health monitoring systems, thereby enhancing the inspection efficiency and operational safety of critical tunnel infrastructure."

Comment 4: The manuscript performs quantitative analysis of crack length, crack width, and leakage area, which represents a valuable engineering-oriented contribution. However, the current crack width estimation method is based on the average width calculated from crack area and length, which may have limited applicability for complex cracks with highly non-uniform widths. It is recommended to discuss the limitations of this approach in the Discussion section. In addition, more advanced width estimation methods, such as local normal-direction width measurement and sub-pixel boundary extraction, could be suggested in the future work section to further improve the reliability of engineering measurements.

Authors' Response:

We thank the reviewer for this constructive suggestion. We agree that the current average-width-based crack width estimation method has limitations for cracks with highly non-uniform width distributions. In response, we have added a discussion of this limitation and future improvements at Section 4.2 where the quantitative measurement results are analyzed. Please refer to Lines 434-440 of the revised manuscript. The specific modifications are as follows:

"The crack width estimation method employed in this study calculates the average width as the ratio of the crack area to its skeleton length. This approach provides a simple and intuitive indicator of the overall crack width, but it may have limited applicability for cracks with highly non-uniform widths, branching structures, or irregular boundaries. In particular, the average width may fail to capture local width variations and critical maximum widths, which are often more relevant to structural safety assessment. Future research will address this limitation by integrating local normal-direction width measurement and sub-pixel boundary extraction techniques to obtain more accurate and reliable crack width measurements.

lines 454-457 The overall language quality of the manuscript is generally acceptable; however, some sentences are relatively lengthy and may affect readability. The manuscript would benefit from further revision with respect to: English grammar and sentence structure; consistency of tense usage; consistency and standardization of technical terminology.

Authors' Response:

We thank the reviewer for the helpful comment on language quality. In response, we have carefully revised the manuscript to improve English grammar, sentence structure, tense consistency, and the standardization of technical terminology. Several lengthy sentences have also been shortened or restructured to enhance readability. All corresponding revisions have been marked in red font in the revised manuscript.

ADDITIONAL NOTES:

We have uploaded the following files as required:

1. Response to Reviewers (this document)

2. Revised Manuscript with Track Changes

3. Manuscript (without track changes)

4. Figure 9

5. Supporting Information files, including S1 Data and S1 Code

We greatly appreciate the time and effort devoted by the editor and reviewers to evaluating our work. We hope that the revised manuscript is now suitable for further consideration by PLOS ONE.

Sincerely,

Qifeng Yu

College of Transport & Communications, Shanghai Maritime University
