# Supplementary material for: Semantic segmentation and quantitative analysis of tunnel cracks and water leakage using a TransUNet framework
Source: PLoS One. 2026 Aug 11;21(8):e0349175. doi: 10.1371/journal.pone.0349175 (PMC13460598; doi:10.1371/journal.pone.0349175)
Supplement: Peer review history

# Peer review: Decision Letter 0

**DOI:** 10.1371/journal.pone.0349175.r001

**Authors:** Baohua Guo

18 May 2026

Dear Dr. Yu,

Please submit your revised manuscript by Jul 17 2026 11:59PM. If you will need more time than this to complete your revisions, please reply to this message or contact the journal office at plosone@plos.org. When you're ready to submit your revision, log on to https://www.editorialmanager.com/pone/ and select the 'Submissions Needing Revision' folder to locate your manuscript file.

- A letter that responds to each point raised by the academic editor and reviewer(s). You should upload this letter as a separate file labeled 'Response to Reviewers'.
- A marked-up copy of your manuscript that highlights changes made to the original version. You should upload this as a separate file labeled 'Revised Manuscript with Track Changes'.
- An unmarked version of your revised paper without tracked changes. You should upload this as a separate file labeled 'Manuscript'.

If you would like to make changes to your financial disclosure, please include your updated statement in your cover letter. Guidelines for resubmitting your figure files are available below the reviewer comments at the end of this letter.

If applicable, we recommend that you deposit your laboratory protocols in protocols.io to enhance the reproducibility of your results. Protocols.io assigns your protocol its own identifier (DOI) so that it can be cited independently in the future. For instructions see: https://journals.plos.org/plosone/s/submission-guidelines#loc-laboratory-protocols. Additionally, PLOS ONE offers an option for publishing peer-reviewed Lab Protocol articles, which describe protocols hosted on protocols.io. Read more information on sharing protocols at https://plos.org/protocols?utm_medium=editorial-email&utm_source=authorletters&utm_campaign=protocols.

As the corresponding author, your ORCID iD is verified in the submission system and will appear in the published article. PLOS supports the use of ORCID, and we encourage all coauthors to register for an ORCID iD and use it as well. Please encourage your coauthors to verify their ORCID iD within the submission system before final acceptance, as unverified ORCID iDs will not appear in the published article. *Only*  the individual author can complete the verification step; PLOS staff *cannot*  verify ORCID iDs on behalf of authors.

We look forward to receiving your revised manuscript.

Kind regards,

Baohua Guo

Academic Editor

PLOS One

Journal Requirements:

When submitting your revision, we need you to address these additional requirements.

1. Please ensure that your manuscript meets PLOS ONE's style requirements, including those for file naming. The PLOS ONE style templates can be found at

https://journals.plos.org/plosone/s/file?id=wjVg/PLOSOne_formatting_sample_main_body.pdf and https://journals.plos.org/plosone/s/file?id=ba62/PLOSOne_formatting_sample_title_authors_affiliations.pdf

2. In the online submission form, you indicated that the data presented in this study is available on request from the corresponding author. The data is not publicly available due to the privacy restrictions of the collaborating organization and safety regulations concerning infrastructure data.

All PLOS journals now require all data underlying the findings described in their manuscript to be freely available to other researchers, either 1. In a public repository, 2. Within the manuscript itself, or 3. Uploaded as supplementary information.

This policy applies to all data except where public deposition would breach compliance with the protocol approved by your research ethics board. If your data cannot be made publicly available for ethical or legal reasons (e.g., public availability would compromise patient privacy), please explain your reasons on resubmission and your exemption request will be escalated for approval.

3. Thank you for stating the following financial disclosure:

This research was funded by the Science and Technology Program of Guangxi, China (No. 2023AB08158)

Please state what role the funders took in the study. If the funders had no role, please state: "The funders had no role in study design, data collection and analysis, decision to publish, or preparation of the manuscript."

If this statement is not correct you must amend it as needed.

Please include this amended Role of Funder statement in your cover letter; we will change the online submission form on your behalf.

4. We note that Figure(s) 1, 2, 3, 4, 7 in your submission contain copyrighted images. All PLOS content is published under the Creative Commons Attribution License (CC BY 4.0), which means that the manuscript, images, and Supporting Information files will be freely available online, and any third party is permitted to access, download, copy, distribute, and use these materials in any way, even commercially, with proper attribution. For more information, see our copyright guidelines: http://journals.plos.org/plosone/s/licenses-and-copyright.

We require you to either (1) present written permission from the copyright holder to publish these figures specifically under the CC BY 4.0 license, or (2) remove the figures from your submission:

a. You may seek permission from the original copyright holder of Figure(s) 1, 2, 3, 4, 7 to publish the content specifically under the CC BY 4.0 license.

We recommend that you contact the original copyright holder with the Content Permission Form (http://journals.plos.org/plosone/s/file?id=7c09/content-permission-form.pdf) and the following text:

“I request permission for the open-access journal PLOS ONE to publish XXX under the Creative Commons Attribution License (CCAL) CC BY 4.0 (http://creativecommons.org/licenses/by/4.0/). Please be aware that this license allows unrestricted use and distribution, even commercially, by third parties. Please reply and provide explicit written permission to publish XXX under a CC BY license and complete the attached form.”

Please upload the completed Content Permission Form or other proof of granted permissions as an "Other" file with your submission.

In the figure caption of the copyrighted figure, please include the following text: “Reprinted from [ref] under a CC BY license, with permission from [name of publisher], original copyright [original copyright year].”

b. If you are unable to obtain permission from the original copyright holder to publish these figures under the CC BY 4.0 license or if the copyright holder’s requirements are incompatible with the CC BY 4.0 license, please either i) remove the figure or ii) supply a replacement figure that complies with the CC BY 4.0 license. Please check copyright information on all replacement figures and update the figure caption with source information. If applicable, please specify in the figure caption text when a figure is similar but not identical to the original image and is therefore for illustrative purposes only.

5. Please note that PLOS One has specific guidelines on code sharing for submissions in which author-generated code underpins the findings in the manuscript. In these cases, we expect all author-generated code to be made available without restrictions upon publication of the work. Please review our guidelines at https://journals.plos.org/plosone/s/materials-and-software-sharing#loc-sharing-code and ensure that your code is shared in a way that follows best practice and facilitates reproducibility and reuse.

6. Please provide a complete Data Availability Statement in the submission form, ensuring you include all necessary access information or a reason for why you are unable to make your data freely accessible. If your research concerns only data provided within your submission, please write "All data are in the manuscript and/or supporting information files" as your Data Availability Statement.

7. Thank you for stating the following in the Acknowledgments Section of your manuscript:

This research was supported by the Royal Academy of Engineering (RAEng) through a research fellowship awarded to N. V.

We note that you have provided funding information that is not currently declared in your Funding Statement. However, funding information should not appear in the Acknowledgments section or other areas of your manuscript. We will only publish funding information present in the Funding Statement section of the online submission form.

Please remove any funding-related text from the manuscript and let us know how you would like to update your Funding Statement. Currently, your Funding Statement reads as follows:

Royal Academy of Engineering (RAEng) Research Fellowship to NV

Please include your amended statements within your cover letter; we will change the online submission form on your behalf.

8. Please update your submission to use the PLOS LaTeX template. The template and more information on our requirements for LaTeX submissions can be found at http://journals.plos.org/plosone/s/latex.

9. We note that Figure(s) 1-9 in your submission contain copyrighted images. All PLOS content is published under the Creative Commons Attribution License (CC BY 4.0), which means that the manuscript, images, and Supporting Information files will be freely available online, and any third party is permitted to access, download, copy, distribute, and use these materials in any way, even commercially, with proper attribution. For more information, see our copyright guidelines: http://journals.plos.org/plosone/s/licenses-and-copyright.

We require you to either (1) present written permission from the copyright holder to publish these figures specifically under the CC BY 4.0 license, or (2) remove the figures from your submission:

a. You may seek permission from the original copyright holder of Figure(s) 1-9 to publish the content specifically under the CC BY 4.0 license.

We recommend that you contact the original copyright holder with the Content Permission Form (http://journals.plos.org/plosone/s/file?id=7c09/content-permission-form.pdf) and the following text:

“I request permission for the open-access journal PLOS ONE to publish XXX under the Creative Commons Attribution License (CCAL) CC BY 4.0 (http://creativecommons.org/licenses/by/4.0/). Please be aware that this license allows unrestricted use and distribution, even commercially, by third parties. Please reply and provide explicit written permission to publish XXX under a CC BY license and complete the attached form.”

Please upload the completed Content Permission Form or other proof of granted permissions as an "Other" file with your submission.

In the figure caption of the copyrighted figure, please include the following text: “Reprinted from [ref] under a CC BY license, with permission from [name of publisher], original copyright [original copyright year].”

b. If you are unable to obtain permission from the original copyright holder to publish these figures under the CC BY 4.0 license or if the copyright holder’s requirements are incompatible with the CC BY 4.0 license, please either i) remove the figure or ii) supply a replacement figure that complies with the CC BY 4.0 license. Please check copyright information on all replacement figures and update the figure caption with source information. If applicable, please specify in the figure caption text when a figure is similar but not identical to the original image and is therefore for illustrative purposes only.

10. Please include captions for your Supporting Information files at the end of your manuscript, and update any in-text citations to match accordingly. Please see our Supporting Information guidelines for more information: http://journals.plos.org/plosone/s/supporting-information.

11. If the reviewer comments include a recommendation to cite specific previously published works, please review and evaluate these publications to determine whether they are relevant and should be cited. There is no requirement to cite these works unless the editor has indicated otherwise.

[Note: HTML markup is below. Please do not edit.]

Reviewers' comments:

Reviewer's Responses to Questions

**Comments to the Author**

1. Is the manuscript technically sound, and do the data support the conclusions?

Reviewer #1: Yes

Reviewer #2: Yes

**********

2. Has the statistical analysis been performed appropriately and rigorously?

Reviewer #1: Yes

Reviewer #2: Yes

**********

3. Have the authors made all data underlying the findings in their manuscript fully available?

The PLOS Data policy

Reviewer #1: Yes

Reviewer #2: Yes

**********

4. Is the manuscript presented in an intelligible fashion and written in standard English?

Reviewer #1: Yes

Reviewer #2: Yes

**********

Reviewer #1: The manuscript presents a semantic segmentation and quantitative analysis framework based on TransUNet for tunnel lining crack and water leakage detection. The study has certain engineering application value, and the overall manuscript is well organized, with a relatively clear experimental workflow and intuitive presentation of results. In particular, the integration of geometric quantitative analysis with engineering validation enhances the practical significance of the study. However, from the perspectives of manuscript standardization and academic presentation, several issues remain regarding language expression, experimental analysis, literature review, and discussion of results. The manuscript is recommended for further revision and improvement. Specific comments are as follows:

1.The manuscript contains several terminology inaccuracies as well as inconsistencies between British and American English usage. The authors are encouraged to carefully proofread the manuscript and unify the writing style throughout. For example:

a)On Page 7, Line 157, “Attenuated Spatial Pyramid Pooling (ASPP)” is incorrect and should be revised to “Atrous Spatial Pyramid Pooling (ASPP)”;

b)Terms such as “lesion,” “pathologies,” and “disease types,” which are more commonly used in medical imaging literature, appear throughout the manuscript and may result from direct translation. It is recommended to replace them with terminology more appropriate for civil engineering contexts, such as “defect types,” “distress categories,” or “tunnel defects”;

c)British and American English spellings are mixed in the manuscript, such as “modelling/modeling” and “whilst/while.” A consistent language style should be adopted;

d)In Fig. 1 and Fig. 2, the term “masked images” is not sufficiently precise and is recommended to be replaced with “ground-truth masks” or “annotation masks.”

2.Although the literature review is generally comprehensive, it remains largely focused on conventional CNN-based approaches, while the discussion of recent Transformer-based methods for infrastructure defect detection is relatively limited. It is recommended that the authors further incorporate recent studies, such as: Applications of SegFormer in infrastructure defect detection; Fine-grained crack segmentation models such as CrackFormer and CrackSegFormer; Research related to lightweight Transformer architectures and boundary enhancement strategies.

3.In the water leakage detection task, U-Net achieves better F1-score and IoU values than TransUNet. However, the manuscript repeatedly emphasizes that TransUNet demonstrates the best overall performance. It is recommended that the authors present the experimental findings in a more objective and balanced manner. For example: Emphasize the advantages of TransUNet in terms of Precision and boundary integrity; At the same time, acknowledge that U-Net demonstrates superior region overlap and overall balanced performance. In addition, overly absolute expressions such as “significantly outperforms” and “best overall performance” should be used more cautiously to improve the credibility and rigor of the discussion.

4.The description of the dataset annotation procedure is relatively brief. It is recommended that the authors provide additional details regarding: Annotation tools and annotation workflow; Annotator information and annotation principles; Whether manual verification and correction were performed. Furthermore, the acquisition process of the Ground Truth data should be clarified.

5.In the crack length calculation formula around Line 196, only the mathematical expression is currently provided, while the physical meanings of the variables and symbols are not sufficiently explained. It is recommended that the authors provide additional clarification regarding the definitions of the variables and the corresponding calculation process to improve readability and completeness.

Reviewer #2: The paper investigates tunnel lining crack and water leakage detection based on a TransUNet semantic segmentation framework and further conducts quantitative analysis of defect geometry. The topic is relevant to intelligent tunnel inspection and infrastructure maintenance, and the study demonstrates practical engineering relevance. The manuscript is generally well organized, and the experimental workflow is clearly presented. In particular, the integration of semantic segmentation with quantitative defect measurement improves the practical applicability of the proposed framework. There are some comments.

1. The manuscript mentions that both public datasets and engineering field images were adopted; however, important information regarding the dataset composition remains insufficiently described. In particular, the proportions of different data sources, annotation procedures, and dataset construction details are not clearly presented. Additional information regarding dataset preparation and annotation is needed to improve the reliability and reproducibility of the study.

2. The experimental evaluation is mainly based on metrics such as Precision, Recall, F1-score, and IoU. While these indicators are commonly used in semantic segmentation tasks, the statistical analysis of the experimental results is still relatively limited. It would be beneficial to further provide: mean and standard deviation (std) obtained from repeated experiments; error distribution analysis; boxplots or error distribution visualizations.

3. Currently, crack length, crack width, and water leakage area are all quantified in pixel units, while the correspondence between image-space measurements and real-world engineering dimensions has not been sufficiently explained. Further clarification regarding the relationship between pixel-scale measurements and actual physical dimensions is recommended to strengthen the engineering applicability of the proposed framework.

4. The manuscript performs quantitative analysis of crack length, crack width, and leakage area, which represents a valuable engineering-oriented contribution. However, the current crack width estimation method is based on the average width calculated from crack area and length, which may have limited applicability for complex cracks with highly non-uniform widths. It is recommended to discuss the limitations of this approach in the Discussion section. In addition, more advanced width estimation methods, such as local normal-direction width measurement and sub-pixel boundary extraction, could be suggested in the future work section to further improve the reliability of engineering measurements.

5. The overall language quality of the manuscript is generally acceptable; however, some sentences are relatively lengthy and may affect readability. The manuscript would benefit from further revision with respect to: English grammar and sentence structure; consistency of tense usage; consistency and standardization of technical terminology.

**********

what does this mean?). If published, this will include your full peer review and any attached files.

If you choose “no”, your identity will remain anonymous but your review may still be made public.

**Do you want your identity to be public for this peer review?**  For information about this choice, including consent withdrawal, please see our Privacy Policy

Reviewer #1: No

Reviewer #2: No

**********

[NOTE: If reviewer comments were submitted as an attachment file, they will be attached to this email and accessible via the submission site. Please log into your account, locate the manuscript record, and check for the action link "View Attachments". If this link does not appear, there are no attachment files.]

To ensure your figures meet our technical requirements, please review our figure guidelines: https://journals.plos.org/plosone/s/figures

You may also use PLOS’s free figure tool, NAAS, to help you prepare publication quality figures: https://journals.plos.org/plosone/s/figures#loc-tools-for-figure-preparation.

NAAS will assess whether your figures meet our technical requirements by comparing each figure against our figure specifications.

# Author comment: Author response to Decision Letter 1

**DOI:** 10.1371/journal.pone.0349175.r002

16 Jul 2026

RESPONSES TO REVIEWER 1

We sincerely appreciate the reviewer’s careful and thorough evaluation of our manuscript. Your insightful comments were extremely valuable in helping us identify and correct several numerical inconsistencies, factual inaccuracies, and language issues, thereby substantially improving the rigor, clarity, and overall quality of the manuscript. Detailed responses to each comment are provided below.

Comment 1: The manuscript contains several terminology inaccuracies as well as inconsistencies between British and American English usage. The authors are encouraged to carefully proofread the manuscript and unify the writing style throughout. For example:

a) On Page 7, Line 157, “Attenuated Spatial Pyramid Pooling (ASPP)” is incorrect and should be revised to “Atrous Spatial Pyramid Pooling (ASPP)”;

b) Terms such as “lesion,” “pathologies,” and “disease types,” which are more commonly used in medical imaging literature, appear throughout the manuscript and may result from direct translation. It is recommended to replace them with terminology more appropriate for civil engineering contexts, such as “defect types,” “distress categories,” or “tunnel defects”;

c) British and American English spellings are mixed in the manuscript, such as “modelling/modeling” and “whilst/while.” A consistent language style should be adopted;

d) In Fig. 1 and Fig. 2, the term “masked images” is not sufficiently precise and is recommended to be replaced with “ground-truth masks” or “annotation masks.”

Authors’ Response:

We thank you for carefully identifying these terminology and language issues. We have systematically revised the manuscript as follows:

(a) In Section 2.2, we have corrected "Attenuated Spatial Pyramid Pooling (ASPP)" to "Atrous Spatial Pyramid Pooling (ASPP)".

(b) Throughout the manuscript, we have replaced medical terminology with civil engineering terms:

In the Introduction, "lesion characteristics" have been changed to "defect characteristics".

In Section 2.3, "lesion pixels" has been changed to "defect pixels".

In Section 3, "disease types" has been changed to "defect types", and "pathologies" has been changed to "defects".

(c) The manuscript has been unified using American English: "whilst" has been replaced with "while" (e.g., in Section 2.1 on data augmentation), and "modelling" has been changed to "modeling" (e.g., in the title of Section 2.2).

(d) In the captions of Figures 1 and 2, "masked images" has been replaced with "ground‑truth masks".

Comment 2: Although the literature review is generally comprehensive, it remains largely focused on conventional CNN-based approaches, while the discussion of recent Transformer-based methods for infrastructure defect detection is relatively limited. It is recommended that the authors further incorporate recent studies, such as: Applications of SegFormer in infrastructure defect detection; Fine-grained crack segmentation models such as CrackFormer and CrackSegFormer; Research related to lightweight Transformer architectures and boundary enhancement strategies.

Authors’ Response:

Thank you for the suggestion to incorporate recent Transformer‑based studies. We have expanded the literature review in the Introduction; please refer to lines 94-100 of the revised manuscript. The specific modifications are as follows:

“Recent studies have further advanced Transformer-based crack segmentation. For instance, CrackFormer-II embeds novel Transformer encoders into a SegNet-like architecture, achieving higher accuracy with substantially fewer computational resources [26]. LiteCrackSeg, a lightweight hybrid CNN–Transformer model, requires only 2.72 million parameters and achieves an inference speed of 56 frames per second, making it suitable for edge deployment [27]. Along this line of research, TransUNet has shown advantages over traditional CNN models in multi-scale feature representation, structural integrity restoration, and small-object detection, thereby offering improved accuracy and robustness for crack segmentation tasks.”

The added related references are as follows:

26. Liu H, Yang J, Miao X, Mertz C, Kong H. CrackFormer network for pavement crack segmentation. IEEE Trans Intell Transp Syst 2023, 24, 9240-9252. https://doi.org/10.1109/TITS.2023.3266776

27. Gobena KA, Rakib MYK, Tesema FB, Asafa GF, Ren S. LiteCrackSeg: A lightweight hybrid CNN–transformer for efficient crack segmentation. PLOS ONE 2026, 21, e0347765. https://doi.org/10.1371/journal.pone.0347765

Comment 3: In the water leakage detection task, U-Net achieves better F1-score and IoU values than TransUNet. However, the manuscript repeatedly emphasizes that TransUNet demonstrates the best overall performance. It is recommended that the authors present the experimental findings in a more objective and balanced manner. For example: Emphasize the advantages of TransUNet in terms of Precision and boundary integrity; At the same time, acknowledge that U-Net demonstrates superior region overlap and overall balanced performance. In addition, overly absolute expressions such as “significantly outperforms” and “best overall performance” should be used more cautiously to improve the credibility and rigor of the discussion.

Authors’ Response:

We thank the reviewer for pointing out the need for a more objective and balanced presentation. We have revised the discussion in Section 3.2 and the Conclusions accordingly; please refer to lines 23-26 (in Abstract), lines 445-448 and lines 454-457 (in Conclusions) of the revised manuscript. The specific modifications are as follows:

“In terms of precision, boundary preservation, and geometric consistency, the proposed method shows clear advantages over the comparison models, while U‑Net exhibits stronger region overlap for water leakage detection. Overall, the method meets the requirements of offline inspection and near-real-time applications.” Please refer to lines 23-26.

“Compared with U‑Net and DeepLabv3+, the proposed TransUNet framework demonstrates advantages in precision, boundary preservation, and crack detection. While U‑Net achieves higher IoU for water leakage, TransUNet provides a more balanced performance across both defect types, together with high inference efficiency, making it a practical choice for tunnel inspection.” Please refer to lines 445-448.

“Segmentation Performance: TransUNet achieves strong performance on both crack and water leakage detection. For cracks, it attains the highest IoU of 71.57% and an accuracy of 98.38%. For water leakage, it yields the highest precision of 91.51%, effectively reducing false positives, whereas U‑Net shows better region overlap with an IoU of 66.69%. Given the engineering priority of minimizing false alarms, TransUNet offers a compelling advantage.” Please refer to lines 454-457.

Comment 4: The description of the dataset annotation procedure is relatively brief. It is recommended that the authors provide additional details regarding: Annotation tools and annotation workflow; Annotator information and annotation principles; Whether manual verification and correction were performed. Furthermore, the acquisition process of the ground truth data should be clarified.

Authors’ Response:

We thank the reviewer for the helpful suggestion regarding the need for a more detailed description of the dataset annotation procedure. In response, we have added a more comprehensive explanation in Section 2.1 of the revised manuscript. Please refer to Lines 126-137 for the corresponding revisions. The specific modifications are as follows:

“Among these images, approximately 70% were obtained from publicly available datasets [30,31], while the remaining 30% were collected from an in-service tunnel. Pixel-wise annotations were generated using LabelMe and Roboflow Annotate following a standardized annotation workflow. Specifically, trained annotators first identified the visible defect regions in each image. For crack defects, the crack centerlines were manually traced and then dilated according to the observed crack width to generate pixel-level crack masks. For water leakage defects, polygonal boundaries were manually drawn along the visible edges of leakage stains. The annotation principle was to include only clearly visible defect areas and to avoid labeling shadows, stains, joints, or texture variations that could not be confidently identified as target defects. After the initial annotation, all masks were manually inspected and corrected by the research team to ensure annotation consistency and reduce subjective errors. The finalized pixel-wise masks were then exported as the ground truth data for model training and evaluation. To maintain consistency during the training process, all images and corresponding Ground Truth masks were resized to 512 × 512 pixels.”

Comment 5: In the crack length calculation formula around Line 196, only the mathematical expression is currently provided, while the physical meanings of the variables and symbols are not sufficiently explained. It is recommended that the authors provide additional clarification regarding the definitions of the variables and the corresponding calculation process to improve readability and completeness.

Authors’ Response:

We sincerely thank the reviewer for highlighting the need for a more detailed explanation. Accordingly, we have supplemented Section 2.4, “Tunnel Distress Measurement Algorithm,” with additional clarification. Please refer to lines 225–228 and lines 232-234 of the revised manuscript for the corresponding revisions. The specific modifications are as follows:

“In Equation (6), the crack skeleton is represented as an ordered sequence of pixel coordinates, denoted as (x_{1},y_{1}), (x_{2},y_{2}), … , (x_{n},y_{n}), where n is the total number of skeleton pixels. The Euclidean distance between each pair of adjacent skeleton points, (x_{i},y_{i}) and (x_{i+1},y_{i+1}) , is calculated. The total crack length L , measured in pixels, is then obtained by summing these distances over all consecutive point pairs from i=1 to n-1.”

“In a two-dimensional pit mask image, each pixel has a constant area of 1 pixel² in the image space. Therefore, the total pixel area 𝐴 of the crack is calculated by summing all foreground pixels in the mask. Here, L denotes the crack length obtained using Equation (6), and the average width ω is also expressed in pixels.”

RESPONSES TO REVIEWER 2

We are very grateful to you for the insightful and constructive suggestions, including the valuable references and recommendations on section structure, which have significantly strengthened our manuscript.

Comment 1: The manuscript mentions that both public datasets and engineering field images were adopted; however, important information regarding the dataset composition remains insufficiently described. In particular, the proportions of different data sources, annotation procedures, and dataset construction details are not clearly presented. Additional information regarding dataset preparation and annotation is needed to improve the reliability and reproducibility of the study.

Authors’ Response:

We thank the reviewer for emphasizing the importance of dataset composition and reproducibility. We have supplemented Section 2.1 with the following information; please refer to lines 126-137 of the revised manuscript. The specific modifications are as follows:

“Among these images, approximately 70% were obtained from publicly available datasets [30,31], while the remaining 30% were collected from an in-service tunnel. Pixel-wise annotations were generated using LabelMe and Roboflow Annotate following a standardized annotation workflow. Specifically, trained annotators first identified the visible defect regions in each image. For crack defects, the crack centerlines were manually traced and then dilated according to the observed crack width to generate pixel-level crack masks. For water leakage defects, polygonal boundaries were manually drawn along the visible edges of leakage stains. The annotation principle was to include only clearly visible defect areas and to avoid labeling shadows, stains, joints, or texture variations that could not be confidently identified as target defects. After the initial annotation, all masks were manually inspected and corrected by the research team to ensure annotation consistency and reduce subjective errors. The finalized pixel-wise masks were then exported as the ground truth data for model training and evaluation. To maintain consistency during the training process, all images and corresponding Ground Truth masks were resized to 512 × 512 pixels.”

Comment 2: The experimental evaluation is mainly based on metrics such as Precision, Recall, F1-score, and IoU. While these indicators are commonly used in semantic segmentation tasks, the statistical analysis of the experimental results is still relatively limited. It would be beneficial to further provide: mean and standard deviation (std) obtained from repeated experiments; error distribution analysis; boxplots or error distribution visualizations.

Authors’ Response:

We thank the reviewer for recommending additional statistical analysis. Due to computational constraints, repeated training runs were not performed in this study. However, we have supplemented the evaluation with an error distribution analysis based on the ten field-test samples, as reported in Tables 6 and 7. Specifically, a boxplot visualization (Fig. 9) and corresponding statistical summaries have been added to Section 4. Please refer to Lines 412–425 of the revised manuscript for the corresponding revisions. The specific modifications are as follows:

“Fig. 9 presents boxplots of the sample-wise relative errors for crack length, crack width, and water leak-age area measurements. For crack length estimation, the mean relative error is 17.56% with a standard deviation of 16.89%, while the median error is 11.68%. The interquartile range extends from 3.81% to 32.03%, indicating moderate variability among the test samples. Crack width estimation shows a relatively higher mean error of 23.87% and a median error of 20.98%, with an interquartile range from 12.76% to 36.22%. This suggests that crack width measurement is more sensitive to local segmentation deviations, especially for thin or irregular cracks. In contrast, water leakage area estimation achieves a much lower mean relative error of 5.45% and a median error of 3.64%, with an interquartile range from 1.66% to 7.10%, demonstrating higher accuracy and stability. Although one outlier is observed in the water leakage area error distribution, the overall error level remains relatively low. These results indicate that area-based measurement is the most stable, followed by crack length estimation, whereas crack width estimation exhibits the largest deviations and remains the most challenging measurement task.”

Fig 9. Boxplots of sample-wise relative errors for crack length, crack width, and water leakage area measurements.

Comment 3: Currently, crack length, crack width, and water leakage area are all quantified in pixel units, while the correspondence between image-space measurements and real-world engineering dimensions has not been sufficiently explained. Further clarification regarding the relationship between pixel-scale measurements and actual physical dimensions is recommended to strengthen the engineering applicability of the proposed framework.

Authors’ Response:

We thank the reviewer for raising this important issue regarding the engineering applicability of the proposed measurement framework. We agree that the relationship between pixel-scale measurements and real-world physical dimensions should be further clarified. In response, we have added two clarifications in the revised manuscript.

First, at the end of Section 2.4, “Tunnel Distress Measurement Algorithm,” we have supplemented the conversion relationship between image-space measurements and physical dimensions. Please refer to Lines 235-239 of the revised manuscript. The specific addition is as follows:

“A calibration factor k (mm/pixel) can be obtained using a reference object with a known physical length placed in the same imaging plane as the tunnel distress, or through camera calibration under fixed acquisition con

Attachment Submitted filename: Response to Reviewers.pdf

# Peer review: Decision Letter 1

**DOI:** 10.1371/journal.pone.0349175.r003

**Authors:** Baohua Guo

26 Jul 2026

Semantic Segmentation and Quantitative Analysis of Tunnel Cracks and Water Leakage Using a TransUNet Framework

PONE-D-26-20664R1

Dear Dr. Yu,

We’re pleased to inform you that your manuscript has been judged scientifically suitable for publication and will be formally accepted for publication once it meets all outstanding technical requirements.

Within one week, you’ll receive an e-mail detailing the required amendments. When these have been addressed, you’ll receive a formal acceptance letter and your manuscript will be scheduled for publication.

An invoice will be generated when your article is formally accepted. Please note, if your institution has a publishing partnership with PLOS and your article meets the relevant criteria, all or part of your publication costs will be covered. Please make sure your user information is up-to-date by logging into Editorial Manager at Editorial Manager® and clicking the ‘Update My Information' link at the top of the page. For questions related to billing, please contact billing support.

If your institution or institutions have a press office, please notify them about your upcoming paper to help maximize its impact. If they’ll be preparing press materials, please inform our press team as soon as possible -- no later than 48 hours after receiving the formal acceptance. Your manuscript will remain under strict press embargo until 2 pm Eastern Time on the date of publication. For more information, please contact onepress@plos.org.

Kind regards,

Baohua Guo

Academic Editor

PLOS One

Additional Editor Comments (optional):

Reviewers' comments:

Reviewer's Responses to Questions

**Comments to the Author**

Reviewer #1: All comments have been addressed

Reviewer #2: All comments have been addressed

**********

2. Is the manuscript technically sound, and do the data support the conclusions?

Reviewer #1: Yes

Reviewer #2: Yes

**********

3. Has the statistical analysis been performed appropriately and rigorously?

Reviewer #1: Yes

Reviewer #2: Yes

**********

4. Have the authors made all data underlying the findings in their manuscript fully available?

The PLOS Data policy

Reviewer #1: Yes

Reviewer #2: Yes

**********

5. Is the manuscript presented in an intelligible fashion and written in standard English?

Reviewer #1: Yes

Reviewer #2: Yes

**********

Reviewer #1: Thanks for your revision, I think the quality of the manuscript has been improved and can be accpeted now

Reviewer #2: The author has made significant revisions and improvements to the paper based on feedback. I suggest accepting this paper.

**********

what does this mean?). If published, this will include your full peer review and any attached files.

If you choose “no”, your identity will remain anonymous but your review may still be made public.

**Do you want your identity to be public for this peer review?**  For information about this choice, including consent withdrawal, please see our Privacy Policy

Reviewer #1: **Yes:**  Gen Li

Reviewer #2: No

**********

# Editor report: Acceptance letter

**DOI:** 10.1371/journal.pone.0349175.r004

**Authors:** Baohua Guo

PONE-D-26-20664R1

PLOS One

Dear Dr. Yu,

I'm pleased to inform you that your manuscript has been deemed suitable for publication in PLOS One. Congratulations! Your manuscript is now being handed over to our production team.

At this stage, our production department will prepare your paper for publication. This includes ensuring the following:

* All references, tables, and figures are properly cited

* All relevant supporting information is included in the manuscript submission,

* There are no issues that prevent the paper from being properly typeset

You will receive further instructions from the production team, including instructions on how to review your proof when it is ready. Please keep in mind that we are working through a large volume of accepted articles, so please give us a few days to review your paper and let you know the next and final steps.

Lastly, if your institution or institutions have a press office, please let them know about your upcoming paper now to help maximize its impact. If they'll be preparing press materials, please inform our press team within the next 48 hours. Your manuscript will remain under strict press embargo until 2 pm Eastern Time on the date of publication. For more information, please contact onepress@plos.org.

You will receive an invoice from PLOS for your publication fee after your manuscript has reached the completed accept phase. If you receive an email requesting payment before acceptance or for any other service, this may be a phishing scheme. Learn how to identify phishing emails and protect your accounts at https://explore.plos.org/phishing.

If we can help with anything else, please email us at customercare@plos.org.

Thank you for submitting your work to PLOS ONE and supporting open access.

Kind regards,

PLOS ONE Editorial Office Staff

on behalf of

Dr. Baohua Guo

Academic Editor

PLOS One
